# Performance and Environmental Assessment of Biochar-Based Membranes Synthesized from Traditional and Eco-Friendly Solvents

**DOI:** 10.3390/membranes14070153

**Published:** 2024-07-11

**Authors:** Abelline Fionah, Isaac Oluk, Laura Brady, Diana M. Byrne, Isabel C. Escobar

**Affiliations:** 1 Department of Chemistry, University of Kentucky, Lexington, KY 40506, USA; akfionah1@uky.edu; 2 Department of Civil Engineering, University of Kentucky, Lexington, KY 40506, USA; isaacoluk@uky.edu (I.O.); dianabyrne@uky.edu (D.M.B.); 3 Department of Chemical and Materials Engineering, University of Kentucky, Lexington, KY 40506, USA; lbrady12@vols.utk.edu

**Keywords:** adsorptive membranes, petroleum-derived solvents, eco-friendly solvents, biochar, life cycle assessment, uncertainty assessment, sensitivity assessment, leaching studies

## Abstract

Water contamination resulting from coal spills is one of the largest environmental problems affecting communities in the Appalachia Region of the United States. This coal slurry contains potentially toxic substances, such as hydrocarbons, heavy metals, and coal cleaning chemicals, and its leakage into water bodies (lakes, rivers, and aquifers) can lead to adverse health effects not only for freshwater bodies and plant life but also for humans. This study focused on two major experiments. The first experiment involved the use of biochar to create a biochar–polysulfone (BC-PSf) flat-sheet multifunctional membrane to remove organic contaminants, and the other major experiment compared eco-friendly (gamma-valerolactone—GVL; Rhodiasolv^®^ PolarClean—PC) and petroleum-derived solvents (i.e., N-methyl-pyrrolidone—NMP) in the fabrication of the biochar–polysulfone membranes. The resulting membranes were tested for their efficiency in removing both positively and negatively charged organic contaminants from the collected water at varying pH values. A comparative life cycle assessment (LCA) with accompanying uncertainty and sensitivity analyses was carried out to understand the global environmental impacts of incorporating biochar, NMP, GVL, and PC in the synthesis of PSf/NMP, BC-PSf/NMP, PSf/GVL, BC-PSf/GVL, PSf/PC, and BC-PSf/PC membranes at a set surface area of 1000 m^2^. The results showed that the addition of biochar to the membrane matrix increased the surface area of the membranes and improved both their adsorptive and mechanical properties. The membranes with biochar incorporated in their matrix showed a higher potential for contaminant removal than those without biochar. The environmental impacts normalized to the BC-PSf/GVL membrane showed that the addition of biochar increased global warming impacts, eutrophication, and respiratory impacts by over 100% in all the membrane configurations with biochar. The environmental impacts were highly sensitive to biochar addition (Spearman’s coefficient > 0.8). The BC/PSf membrane with Rhodiasolv^®^ PolarClean had the lowest associated global environmental impacts among all the membranes with biochar. Ultimately, this study highlighted potential tradeoffs between functional performance and global environmental impacts regarding choices for membrane fabrication.

## 1. Introduction

Food, water, and energy are three of the most important necessities to sustain life on earth [1]. They are interconnected, where a surplus in one leads to a surplus in the other and vice versa [2,3]. Because of this, they were included in the United Nation’s Sustainable Development Goals as 1: zero hunger; 6: clean water and sanitation; and 7: affordable and clean energy [4].Various nexuses have been developed to address these goals and of them, the nexus for food, energy, and water specifically focuses on finding more sustainable and creative options to address issues with water, food, and energy quality as well as quantity [1]. From biofuels to bio-oils, bio-based products have come to be forefront [5,6,7]. Furthermore, the byproducts of these processes have the potential to be utilized to address further the environmental concerns associated with global civilization and urbanization [6]. Biochar, a byproduct of bio-oil production, can be utilized as an energy source to produce heat energy, in agriculture as fertilizer, and in water treatment as an adsorbent of organic contaminants [8]. This makes biochar an ideal candidate for innovations at the nexus of food, energy, and water systems (INFEWS).

Researchers have heavily studied biochar as an environmentally friendly alternative for numerous environmental remediation applications [9]. This is because biochar is biowaste and its use is therefore considered to have environmental and economic benefits [8]. It can be described as a stable carbon-rich substance that can be obtained as a byproduct of organic matter/biomass pyrolysis [9,10]. The high surface area, functional groups, pH, and high porosity of biochar make it ideal for the removal of organic contaminants, such as dyes, from water [10]. This is because they provide sufficient binding sites for these contaminants. It has also been shown that when biochar is incorporated into the membrane matrix, the mechanical and thermal stability of the membrane can be improved [11]. Biochar is derived from biomass combustion via pyrolysis (fast or slow) as well as gasification [10]. This is carried out in either the absence of oxygen for pyrolysis or very low oxygen supplies for gasification processes at temperatures below 700 °C [8,10]. Pyrolysis, which can be divided into types of very slow, conventional, fast, and flash, yields biochar at different weight percentages [12]. Slow pyrolysis is the preferred form of biochar production as it yields a higher percentage of biochar (~35.0% from dry biomass weight) while producing ~30% bio-oil; furthermore, it can reduce the effects of heavy metals into less toxic byproducts while eliminating pathogens [10,13]. Fast pyrolysis, on the other hand, produces mostly bio-oil as its final product (~70%) while biochar is a byproduct at ~12% dry weight [14,15]. The properties of the biochar produced from pyrolysis, such as surface area, functional groups, hydrophobicity, stability, zeta potential, and pH, depend on the type of raw material (biomass) and the pyrolysis temperature used [14]. Some of the most common biomass feedstocks utilized include agricultural wastes, algae biomass, crop residues, animal wastes, activated sludge, energy crops, and digestate [16,17].

Most of the biochar from bio-oil production is utilized in heat production and what is left is typically used as fertilizer or for soil improvements [8]. There have been limited studies that have shown the application of biochar for water remediation. For example, Ghaffar et al. synthesized membrane composites with biochar made from wood feedstock blended with polyvinylidene fluoride (PVDF). The biochar composite membranes showed high adsorption capacities to rhodamine B (RhB) dye as well as high retention for *E. coli* [18]. With RhB dye being positively charged, this demonstrated that the composite membranes could absorb positively charged particles/contaminants. Wang et al. successfully showed that the addition of biochar into a membrane reactor facilitated the nitrogen removal from municipal wastewater and reduced the fouling rate of the membranes [19]. These works emphasize that the high surface area of biochar is beneficial for water treatment performance because the added adsorption sites aid in the removal of contaminants.

Another approach that many researchers have utilized to address issues within the food–energy–water nexus is the use of membrane separations. Here, many advanced modifications have been carried out for improved membrane performance in the removal of organic contaminants such as organic dyes, algal toxins, etc., from water. Some examples of such modifications include immobilization of nanoparticles onto the membrane surface [20], introduction of surface charge, formation of composites [21], and development of green membrane detection methods [22], among others. However, the use of traditional membranes has been criticized due to the need for harsh petroleum-derived solvents, which have associated health and environmental concerns. Membranes are mostly made using petroleum-derived solvents, such as tetrahydrofuran (THF), N,N-dimethylformamide (DMF), and 1-methyl-2-pyrrolidone (NMP), which have been classified as toxic, carcinogenic, and persistent; additionally, these solvents can bioaccumulate, which has led to some of them being banned in many parts of the world like Europe [23]. Therefore, the push for greener nontoxic solvents is at an all-time high [24]. Ideally, a green solvent is nontoxic, biodegradable, recyclable, inexpensive, non-volatile, and produced from renewable sources [25,26]. To address this concern, some solvents, such as γ-valerolactone (GVL) and Rhodiasolv^®^ PolarClean (PC) have emerged as promising [27]. GVL is considered eco-friendly and nontoxic and can be obtained from acid hydrolysis of cellulose-based biomass (wood) [28,29]. Since it has a lactone-based structure that is similar to NMP, it has been used extensively in the literature as a greener alternative to NMP solvent [24,28,30]. Rhodiasolv^®^ PolarClean, on the other hand, is a green solvent with high solvency that is industrially produced through the valorization of 2-methyleglutaronitrile (MGN), which is a byproduct of Nylon-66 production [31]. This byproduct would otherwise be burned [27]. These solvents have shown potential for the replacement of petroleum-derived solvents [27]. In a paper by Rasool and Vankelecom, membranes based on cellulose acetate (CA), polyimide (PI), cellulose triacetate (CTA), polyethersulfone (PES), and polysulfone (PSU) were synthesized by utilizing GVL as a bio-based green solvent via non-solvent-induced phase separation (NIPS) process with permeance of rose Bengal (RB) rejection over 90% [24]. To improve the properties of PolarClean and GVL as well as control the pore structure of the resulting membranes, Dong et al. combined both PolarClean and GVL in equal amounts as cosolvents for the synthesis of PSf membranes for water treatment. The synthesized membranes exhibited similar performance in water filtration as those synthesized with petroleum-derived membranes [32].

Sustainable design tools such as life cycle assessment (LCA) can aid in understanding and addressing potential global environmental impacts connected with a product or system throughout its lifetime [33,34,35]. LCA is carried out in accordance with the International Standards (ISO) 14040 and 14044 frameworks [33,36,37], which include four steps—goal and scope definition, life cycle inventory analysis (LCI), life cycle impact assessment (LCIA), and life cycle interpretation [36,37]. Present system designs, processes, and operations of membrane systems for water infrastructure pose sustainability as a grand challenge [35,38,39,40,41,42]. As a result, recent research on membrane technology is increasingly incorporating LCA [43]. For instance, Yadav et al. evaluated the environmental impacts of fabricating 1000 m^2^ of hollow fiber polymeric membranes [35]. Their study considered membrane materials including fossil-based and bio-based polymers (polysulfone, polyvinylidene fluoride, and cellulose acetate), traditional solvents (e.g., N-Methyl-2-pyrrolidone and N-dimethylformamide), and an alternative green solvent (ethylene carbonate, EC). They revealed that replacing a fossil-based with a bio-based polymer resulted in a minimal effect on associated global environmental impacts [35]. Conversely, using a green solvent instead of a traditional solvent significantly reduced the overall environmental impact of the fabricated membranes by up to 35% [35,38]. The global environmental impacts of green solvent production remain unknown, as its production via a toxic process can negatively affect the sustainability of membrane production [35,38]. Additionally, while comprehensive research has been conducted on the performance of biochar and its applications in water treatment [41,42,44,45], current research has not evaluated the performance and environmental impacts of multifunctional adsorptive membranes fabricated from incorporating biochar into a polysulfone (PSf) membrane matrix in the presence of eco-friendly solvents (e.g., PolarClean).

Though there has been some research focused on biochar-based membranes [11,18], there have been no studies that focused on incorporating biochar into a polysulfone (PSf) membrane matrix in the presence of green solvents, such as PolarClean, to fabricate multifunctional adsorptive membranes. Furthermore, there are limited LCA studies that focus on membrane from cradle to gate using green solvents [11]. Herein, biochar-based membranes have been synthesized and their adsorptive properties have been investigated for the removal of both positively and negatively charged organic dyes. Leaching studies were carried out to understand the fate of the biochar within the membrane matrix. Furthermore, LCA was performed along with uncertainty and sensitivity analyses to understand the global environmental impacts of the synthesized membranes. This study was based on the hypothesis that the addition of biochar into the membrane matrix would increase the adsorptive properties of PSf membranes while improving their mechanical properties and that using eco-friendly solvents would significantly reduce the global environmental impacts of these membranes.

## 2. Materials and Methods

### 2.1. Materials

N-methyl-2-pyrrolidone (NMP), bovine serum albumin (BSA), ACS-grade hydrochloric acid (HCl), methyl orange dye, and methylene blue dye were obtained from VWR international (Solon, OH, USA). γ-valerolactone (GVL) was obtained from Sigma-Aldrich (St Louis, MO, USA), Rhodiasolv^®^ PolarClean was provided by Solvay (Princeton, NJ, USA), and biochar was obtained from Fisher Scientific (Waltham, MA, USA).

### 2.2. Characterization of Solid Biochar

#### 2.2.1. Adsorption Experiments for Isotherms

To determine the best adsorption mechanism and maximum adsorption capacity of the biochar, methylene blue (MB) dye (a cationic dye with a very prevalent intense absorption peak at around 664 nm in the UV–visible spectra, making it easy to quantify [46]) was used for quantitative analysis and various isotherms were modeled. The procedure utilized was adopted from the literature [47] and modified as follows: Two mL of methylene blue dye pH 3.14 at concentrations ranging from 0 ppm to 1000 ppm were added to 25 mg of biochar. The solutions were kept in an Innova 4000 incubator shaker (Edison, NJ, USA) maintained at 30 °C at 100 rpm for 48 h. This was repeated and tripled for pH values of 5.19 and 10.92. Then, the concentration was analyzed using the VWR^®^ UV-6300 PC double-beam spectrometer (Radnor, PA, USA), and the adsorption capacity was calculated using the adsorption capacity equation (Equation (1)) [47,48],

(1) $$q=\frac{C_{o}-Ce}{m}V$$

where *q* is the adsorption capacity in mg/g, *C_0_* is the initial concentration of methylene blue dye in ppm, *C_e_* is the final dye concentration in ppm, *V* is the volume of dye used, and m is the mass of biochar.

Modeling of the isotherms was carried out using the Langmuir, Freundlich, and Sips models due to their ease of use and popularity [49,50]. These were compared to experimental data. Experimental data were fitted using both pseudo 1st-order and pseudo 2nd-order nonlinear kinetic models. Equations for the selected models are listed below.

Langmuir isotherm [47,49] (Equation (2)) shows the equilibrium between adsorbate and substrate, where adsorption is limited to one molecular layer [51]. In this model, four general assumptions are made, namely, no lateral interaction between the adsorbed molecules, monolayer adsorption is observed, presence of homogeneous active sites, and finally, availability of constant adsorption energy. The equilibrium factor (RL) shows the feasibility of the adsorption. If the RL is greater than 1, adsorption is favorable; if it is less than 1, it is unfavorable; if it is equal to 1, it is linear; and if it is close or equal to zero, adsorption is irreversible [52].

(2) $$q=\frac{q_{m}Ce}{Kd+Ce}$$

where *q* is the adsorption capacity in mg/g, *q_m_* is the maximum adsorption capacity in mg/g, *C_e_* (mg/L) is the final dye concentration in mg/L or ppm, and *K_d_* is the Langmuir adsorption equilibrium constant in L/g.

In the Freundlich mathematical isotherm [47,50,53] (Equation (3)), surface heterogeneity is accounted for. This can be caused by either multilayer adsorption or exponential distribution of adsorbent active sites [52,53]. Though this model can explain the behavior of the adsorbent to some extent, it has limited applicability as it can only account for a limited concentration range before it becomes linear. Additionally, the Freundlich constant *K* is temperature-dependent [53].

(3) $$q=KCe^{1/n}$$

where *q* is the adsorption capacity in mg/g, *K* is the Freundlich constant in L/mg, *C_e_* is the final dye concentration in mg/L, and n is the heterogeneity factor.

Lastly, the Sips model [47,49,54] (Equation (4)) combines both the Freundlich and the Langmuir equations and addresses their limitations. It projects a high concentration limit, describes monolayer adsorption, and can also be used in heterogeneous systems. Because of this, it follows the Freundlich model at low concentrations and the Langmuir model at high concentrations [52].

(4) $$q=\frac{q_{m}K_{s}Ce^{1/ns}}{1+K_{s}Ce^{1/ns}}$$

where *q* is the adsorption capacity in mg/g, *q_m_* is the maximum adsorption capacity in mg/g, *K_s_* is the Sips equilibrium constant in mg/L, *C_e_* is the final dye concentration in mg/L, and 1/ns is the heterogeneity factor.

#### 2.2.2. Kinetic Adsorption Models

For adsorption kinetics, time-dependent adsorption experimental data is often modeled via both the pseudo 1st-order and pseudo 2nd-order kinetics models [55]. In the pseudo 1st-order model, information about both kinetics and equilibrium can be obtained [55]. Kinetics assumes that the rate of adsorption is directly proportional to time. This is mostly limited to the initial times of adoption [55,56]. The equation for the pseudo 1st-order model can be seen in Equation (5). The pseudo 2nd-order assumes that the adsorption rate is dependent on the adsorption capacity as opposed to the concentration; thus, the rate-limiting step is the chemical adsorption [47,56]:

(5) $$q=q_{m}\left(1-e^{-K_{1}t}\right)$$

where *q_m_* is the maximum adsorption capacity in mg/g, *K*_1_ is the rate constant in (min^−1^), and *t* is the time in min.

The pseudo 2nd-order model follows Equation (6) [47,57]:

(6) $$q=\frac{{q_{m}}^{2}K_{2}t}{1+qmK_{2}t}$$

where *q_m_* is the maximum adsorption capacity in mg/g, *K*_2_ is the rate constant in mg g^−1^ min^−1^, and *t* is time in minutes.

#### 2.2.3. Brunauer–Emmett–Teller (BET) Surface Area Analysis

BET analysis was modified from the literature [58]. Biochar, in an amount of 105.5 mg, was degassed in a glass cell for 8 h for water and contaminant removal. Samples were placed in glass cells to be degassed and analyzed using the BET machine. Glass rods were placed within the cell to minimize the dead space in the cell. After degassing, the cell and its contents were transferred to the analysis port of the micromeritics TriStar 3000 BET analyzer (Norcross, GA, USA). Liquid nitrogen was utilized for temperature control of the sample. Nitrogen gas was then injected into the sample cell along with a calibrated piston and the measurements were obtained.

### 2.3. Membrane Synthesis

Polymer dissolution and membrane formation were adapted from the literature [21,59]. For biochar-containing membranes, biochar granules were dissolved in various solvents in a flask at a constant temperature of 80 °C and intermittent sonication for full dissolution. The components were stirred for varying numbers of days (ranging from 2 to 8 days) to obtain a homogeneous dope solution. The weight fraction of the polymer was 17%, the weight fraction of the solvents was 83%, and the total weight fraction of the biochar additive within the polymer was 2%. The solution was then degassed for 1 h. before membrane casting to remove any associated bubbles. The non-solvent-induced phase separation method (NIPS) was used for membrane formation. The NIPS method is ideal for the formation of flat-sheet polymeric membranes [60]. It involves casting the dope solution onto a substrate and then transferring the casted membranes with the substrate into a non-solvent bath where the solvent migrates from the polymeric matrix to the non-solvent bulk, while the non-solvent bulk enters the matrix through the created channels and is exchanged with the solvent [61]. The dope solution was cast on a glass plate using a doctor blade. The glass and the cast membrane were then transferred to a water bath maintained at room temperature immediately after membrane casting. The water bath acts as a non-solvent, which allows for mixing and de-mixing to occur [60]. Finally, the resulting membranes were stored in DI water for the removal of residual solvent. The specified conditions for each membrane formed can be seen in Table S1.

### 2.4. Membrane Characterization

#### 2.4.1. Thermogravimetric Analysis (TGA)

To determine the thermal stability of the as-synthesized membranes, thermogravimetric analysis studies were carried out on the membrane samples with an average weight of 12 mg using a TA Instruments TGA 550 (New Castle, DE, USA) with a heating rate of 10 °C/min from 25 °C until 1000 °C. The experiments were carried out under a nitrogen atmosphere with a flux of 10–20 mL/min.

#### 2.4.2. Scanning Electron Microscopy (SEM)

The morphology of the PSf ultrafiltration membranes and BC-PSf composite membranes was investigated using an SEM Quanta FEG 250, FEI/ThermoFisher Scientific, (Hillsboro, OR, USA). The samples were assembled using cryofracture for the cross-sections and they were coated with platinum under vacuum before SEM images were taken.

#### 2.4.3. Fourier Transform Infrared (FTIR)

A Thermo Scientific Nicolet iS50 Fourier transform infrared (FTIR) spectrometer was utilized to collect FTIR spectra of both the as-synthesized membranes as well as solid biochar by evaluating the absorbance after 64 scans in the attenuated total reflectance mode. Membranes were air-dried for 24 h before measurements were carried out.

#### 2.4.4. Contact Angle

To understand the wettability of the synthesized membranes, contact angle measurements were obtained using the Krus drop-shape analyzer DSA1005 (Matthews, NC, USA). Using the sensile drop method, a 1 µL of droplet volume was utilized per membrane and the contact angle was measured immediately after the dropping and at intervals of 10 s. Before the measurements, the membranes were completely rinsed with DI water and dried at room temperature overnight. The measurements were triplicated for reproducibility.

#### 2.4.5. Mechanical Strength

To measure the largest force that the membranes could withstand before breaking, mechanical strength tests were carried out. The mechanical properties of the as-synthesized membranes were analyzed using the Instron tensile testing machine 2716-010 (Norwood MA, USA) operating at a max load of 5N and temperature range of −70 °C to 250 °C. the membranes were fully dried before the tests and each test was triplicated for reproducibility.

#### 2.4.6. Zeta Potential

To understand the surface charge of the as-synthesized membranes, the zeta potential was deduced by experimental measurements of electrical resistance in the membrane pores, carried out using the Anto Parr Surpass 81611461 (Graz, Austria). KCl solutions of 0.1 M were utilized as the electrolyte, and pH adjusting was carried out using HCl and NaOH solutions. The KCl solution was used to provide the excess ions to be used to calculate the zeta potential by accounting for the electrostatic interaction between the solid surface, the counter-ions, and the co-ions in the solution [62]. This was carried out for each dried membrane at pHs of 3, 6, and 10. Streaming potential measurements were carried out and zeta potential values were deducted.

#### 2.4.7. X-ray Photoelectron Spectroscopy (XPS) and Depth Profile

The composition of the synthesized composite membranes’ top layers was studied with Thermo-Scientific K-Alpha X-ray photoelectron spectroscopy (Waltham, MA, USA). Depth profile analysis with ion beam trek etching was also performed to show biochar deposition in the pores.

### 2.5. Performance Analysis Flux and Rejection

The performance analysis of both the biochar and the biochar-based membranes was performed in the presence of organic dyes methylene blue, methyl orange, and monomeric protein bovine serum albumin (BSA). This was carried out in various environments where temperature and pH were modified.

For dye adsorption studies investigating the effect of pH on solid biochar, 25 mg of biochar was added to solutions containing the dyes at pHs 3, 6, and 10. Samples were taken periodically for 24 h and then analyzed using UV-VIS (UV-6300PC, Leuven, Belgium). The same was repeated for the investigation of the effect of temperature on biochar adsorption properties. The dyes were kept at temperatures of −4 °C (cold), 23 °C (RT), and 90 °C (hot), and the samples of dye solutions were taken and analyzed for adsorption via UV-Vis over a range of 24 h.

The adsorptive properties of the biochar-based membranes were investigated in two ways. First, by soaking the membranes in solutions of both dyes and BSA solutions, and second, via dead-end filtration. Membranes were soaked in solutions containing 100 ppm of methylene blue, methyl orange dyes, and BSA protein, and samples were taken and analyzed after 24 and 48 h. For filtration studies, pre-compaction was first carried out to flush out contaminants from the membranes; then, 10 ppm methylene blue dye solutions were filtered through the various membranes and the filtrate was collected and analyzed.

### 2.6. Leaching Studies

To understand the stability of biochar within the various membranes, leaching studies were carried out via sitting/soaking, and filtration. Under sitting/soaking experiments, the membranes were soaked in DI water for 10 days, and samples were taken and analyzed for total organic carbon (TOC) using the TA-550 TOC analyzer (New Castle, DE, USA) at varying times. For filtration experiments, DI water was filtered through each membrane for 15 min. Five filtrations were performed for each membrane and samples were analyzed for TOC.

### 2.7. Life Cycle Assessment (LCA)

**Figure 1:**
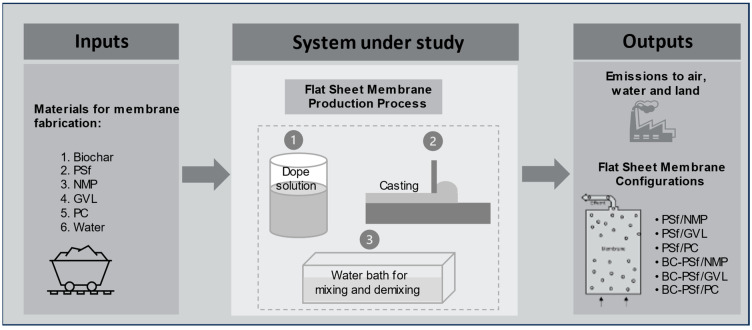
Framework for system analysis and LCA.

Life cycle assessment was performed on biochar-based membranes synthesized from two green solvents (gamma-valerolactone or GVL, and Methyl-5-(dimethylamino)-2-methyl-5-oxopentanoate, Rhodiasolv^®^ PolarClean or PC), and a traditional solvent (i.e., *N*-methyl-2-pyrrolidone or NMP). The use of biochar for creating biochar–polysulfone (BC-PSf) flat-sheet membranes was investigated, and the system framework of this study is summarized in Figure 1. In total, three pairs of flat-sheet membranes with each having two configurations of polymer–solvent and biochar–polymer–solvent (PSf/NMP, BC-PSf/NMP; PSf/GVL, BC-PSf/GVL; and PSf/PC, BC-PSf/PC) were evaluated for global environmental impacts. Foreground data for the LCA were based on the laboratory-scale experiments and background data (for the life cycle inventory) were based on the literature and the Ecoinvent 3.5 inventory database. MATLAB R2023b was used to build a life cycle inventory and carry out a life cycle impact assessment (computational structure described in Section SI3 of the Supplementary Materials) to evaluate the environmental impacts of the membrane configurations under uncertainty. Microsoft^®^ Excel^®^ for Microsoft 365 MSO (Version 2406 Build 16.0.17726.20078) 64-bit and SigmaPlot 15 software were used to organize data and generate graphical representations, respectively.

The goal of the LCA was to compare the global environmental impacts of producing biochar-based polymeric flat-sheet membranes synthesized from various solvents at the laboratory scale level. The system boundary was cradle-to-gate, including all processes for membrane fabrication until the point of application for water treatment [34]. All the relevant raw materials, energy, utilities (e.g., water), chemicals, and emissions involved at the production stage were within the system boundary. Environmental impacts due to the transportation of the materials, electricity used to operate the laboratory and equipment during experiments, the production of machinery, and the plant for flat-sheet membrane fabrication were not considered. Electricity used for the operation of laboratory equipment was excluded due to the inability to allocate consumption to individual projects. Similarly, as with transportation requirements for materials, it was assumed that electricity consumption remained relatively consistent to meet the production needs of all the design configurations and was therefore excluded. The production of a 1000 m^2^ flat-sheet membrane was taken as the functional unit. The lab scale results for 1 m^2^ were scaled linearly to this functional unit such that all inputs (materials and energy) and outputs (emissions) are evaluated on a per functional unit basis.

#### 2.7.1. Life Cycle Inventory (LCI)

Ecoinvent was accessed through SimaPro v.9.0.0.49 Ph.D. modeling software to obtain LCI data from various sectors such as energy production, transport, building materials, and chemical production, among others. Foreground data (Table S2 of the Supplementary Materials) from the laboratory-scale fabrication of a 1 m^2^ flat-sheet membrane was used while background data were sourced from the literature and life cycle inventory databases (Tables S3–S8 of the Supplementary Materials) [40,41,42]. The biochar is produced by slow pyrolysis of wood logs using a kiln-based technology, and the associated inventory data for biochar powder were sourced from studies conducted by Smebye et al. (2017) and Shaheen et al. (2022) (Table S3 of the Supplementary Materials). By percentage volume, the biochar, polymer, and solvent combinations constituted up to 1%, 44–47%, and 53–55% of the flat-sheet membrane, respectively (Table 1).

#### 2.7.2. Life Cycle Impact Assessment (LCIA)

Life cycle impact assessment (LCIA) was conducted using the Tool for the Reduction and Assessment of Chemicals and Other Impacts (TRACI) 2.1 version 1.05 developed by the U.S. Environmental Protection Agency [63] and implemented using SimaPro [64,65]. TRACI includes ten environmental impact categories: ozone depletion (kg CFC-11 eq), global warming (kg CO_2_ eq), acidification (mol H+ eq), eutrophication (kg N eq), smog (kg O_3_ eq), respiratory effects (kg PM_2.5_ eq), carcinogenic (CTUh), non-carcinogenic (CTUh), ecotoxicity (kg 2,4-dioxane eq), and fossil fuel depletion (MJ surplus) [63].

#### 2.7.3. Uncertainty and Sensitivity Analyses

To provide a more robust assessment of the environmental impacts, an uncertainty analysis using 10,000 Monte Carlo simulations was conducted. Monte Carlo offers a probabilistic approach to uncertainty analysis by generating numerous parameter values through random sampling from specified distributions. The material quantities employed in the fabrication process were varied by ±30% to establish uniform distributions and, consequently, output distributions for modeled environmental impacts. Additionally, sensitivity analysis was performed for all material quantities using Spearman’s rank correlation. This analysis facilitated the quantification of relationships between the effect of varied material quantities on the resulting environmental impacts of the fabricated membranes.

## 3. Results and Discussion

### 3.1. Biochar Characterization

#### 3.1.1. Adsorption Isotherms

It has been reported in the literature that pH is one of the major factors affecting the adsorption capacities of biochar [66]. The functional groups in biochar that contain oxygen are pH-dependent. Because of this, the pH of the methylene blue dye solution affects its adsorption onto the biochar and the mechanism by which it adsorbs [67]. When there is an increase in pH, the functional groups are deprotonated. This increases cationic contaminants, attractive forces predominate, and enhances adsorption properties. When the pH decreases, electrostatic repulsion forces predominate, which can lead to diminished adsorption properties as there is competition between the protons and the cations of the solution [68,69]; hence, a pH of 6, close to neutral, is ideal for the adsorption experiments, as there is no competition for active sites. Various adsorption isotherms were then modeled to fit the experimental data.

**Figure 2:**
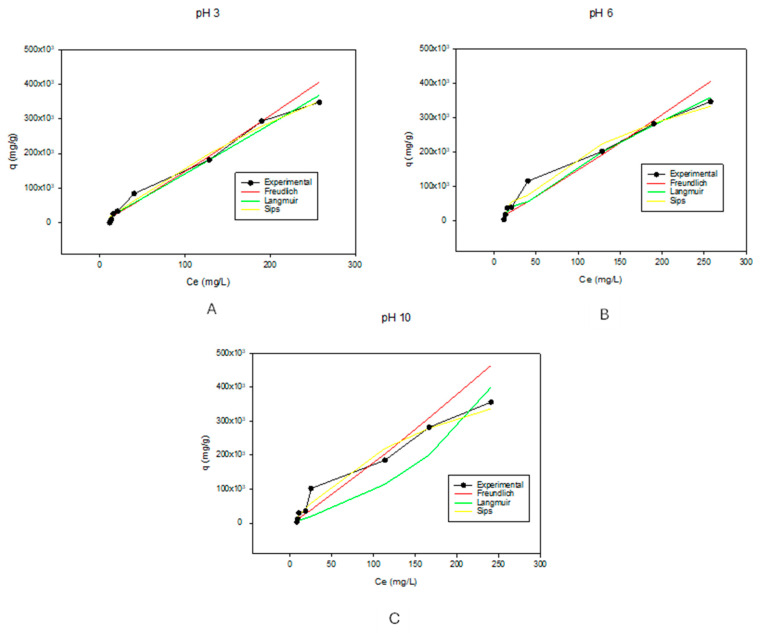
Graphs showing the adsorption isotherm models at pHs of 3, 6, and 10, which correspond to (**A**), (**B**), and (**C**), respectively. For better visualization of the data, the graphs were not started at the 0.0 point.

Table 2 shows the parameters for the adsorption isotherms. These isotherms were calculated and fitted to the Freundlich, Langmuir, and Sips models at different pH values. The R^2^ values show that while in acidic and basic environments, Freundlich isotherms best described the experimental results, while at pH of 6, Langmuir isotherms predominated. The obtained R^2^ values for Freundlich Isotherms were 0.9337, 0.8615, and 0.8238 for pH of 3, 6, and 10, respectively. Applying the Langmuir isotherm model, the R^2^ values were 0.7495, 0.9534, and 0.8202 for pHs 3, 6, and 10, respectively. The Sips model gave R^2^ values of 0.8760, 0.9482, and 0.7091 for pHs 3, 6, and 10, respectively. For the isotherm model to be highly applicable, the correlation values (R^2^ values) must be greater than 0.9 [70]. Because of this, at a pH of 3, the Freundlich isotherm model best described the adsorption behavior of the biochar. As observed in Figure 2A, the Freundlich model aligns with the experimental data on more points than the other models. Furthermore, it had an n-value (which describes the adsorption intensity or surface heterogeneity [52]) greater than 1, which indicated favorable adsorption. The larger the n value, the stronger the interaction between the substrate and adsorbent [71]. Ideally, for favorable adsorption, n-values should be between 1 and 10 [72]. This further shows why the Freundlich adsorption isotherm model predominated at pH values of 3 and 10. For pH 6, the Langmuir model best describes the experimental data with a high correlation value (R^2^). The Langmuir isotherm model indicated that there was monolayer adsorption of the MB dye at this pH. The maximum adsorption capacity (q_m)_ calculated from the Langmuir isotherm model was 1,764,145.7 mg/g. This value was close to the experimental values, indicating that the Langmuir isotherm model was the best fit for the experimental data. For the experimental data at a pH of 10, no R^2^ value was above 0.9 of the investigated models; however, the Freundlich isotherm model came closest with the highest R^2^ value of 0.8238. Despite the correlation value for the pH 10 being less than 0.9, it had an n value greater than 1, which showed that it was a favorable model for the experimental data fitting. Looking at the graph in Figure 2C, the experimental data showed that the value exponentially increased at the lower Ce values before linearizing at the higher values, hence the Freundlich isotherm model. This indicated that there could have been multilayer adsorption of the dye at these pH values [52].

#### 3.1.2. Kinetics Models

**Figure 3:**
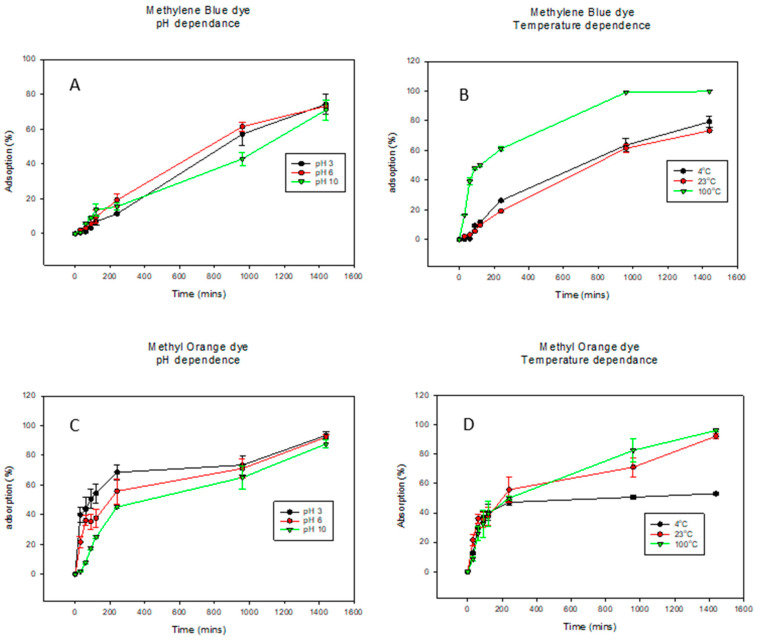
Graphs showing the percent of dye adsorbed over time under varying pHs and temperatures. Methylene blue dye graphs are (**A**) for pH dependence and (**B**) for temperature dependence while methyl orange dye graphs are (**C**) for pH dependence and (**D**) for temperature dependence. For better visualization of the data, the graphs were not started at the 0.0 point.

Kinetic studies are crucial for understanding the adsorption mechanisms and the rate-limiting steps of the reactions [73]. The adsorption of methylene blue dye (MB) onto the biochar (BC) was conducted at pHs 3, 6, and 10 as well as temperatures 4 °C, 23 °C, and 100 °C (Figure 3 and Table 2). For MB dye adsorption (pH dependence curves shown in Figure 3A), pH values of 3, 6, and 10 exhibited similar trends. Here, a pH of 6 shows the highest adsorption while a pH of 10 shows the lowest at about 1000 min. However, given the errors, they ultimately are the same. For the temperature dependence of MB dye adsorption in Figure 3B, while 4 °C and 23 °C exhibit similar trends, at the increased temperature of 100 °C, there was a significant increase in dye adsorption. Similar trends were observed for the pH dependence of adsorption of MO dye in Figure 3C. Interestingly, for MO temperature adsorption, there was a direct relationship between dye adsorption and temperature as the percentage of dye adsorbed increased with increasing temperature in Figure 3D. The results from Figure 3 indicated that compared to pH, temperature had a bigger effect on the adsorption properties of BC. The literature has shown that dye adsorption onto BC increases rapidly at earlier stages and eventually slows down as the active sites of the BC decrease [73,74]. This was also observed in this study in both MB and MO adsorption. In this study, the pseudo first-order and pseudo second-order kinetic models were applied to adsorption kinetics data for the adsorption of MB dye onto BC, as observed in Table 3. The pseudo first-order model was the best fit to explain the kinetic data across all the pHs. It showed the highest correlation values (R^2^) of 0.9885, 0.9958, and 0.9861 for pH values of 3, 6, and 10, respectively, as well as the lowest k values. Accounting for the assumptions of the pseudo first-order kinetic model, adsorption occurred through diffusion through the interface; hence, the rate of adsorption was proportional to the number of available active sites on the BC surface [56,75]. For temperature dependence, the pseudo first-order kinetic model fits the kinetic data better with R^2^ of 0.9981 and 0.9958 for temperatures 4 °C and 23 °C, respectively. However, at elevated temperatures of 100 °C, the pseudo second-order model fits the kinetic model better with an R^2^ value of 0.9964. This indicated that at lower temperatures, the amount of available active sites on the biochar was the limiting step in the MB dye adsorption, while at higher temperatures, the rate-limiting step was the chemical adsorption [73]. The models were fitted to the experimental data and can be seen in Supplemental Information Figures S1 and S2 for pH dependence and temperature dependence, respectively.

The results from Figure 3 indicate that compared to pH, temperature had a bigger effect on the adsorption properties of BC. The literature has shown that dye adsorption onto BC increases rapidly at earlier stages and eventually slows down as the active sites of the BC decrease [73,74]. This was also observed in this study in both MB and MO adsorption. In this study, the pseudo-1st order and pseudo-2nd order kinetic models were applied to adsorption kinetics data for the adsorption of MB dye onto BC, as observed in Table 4. The pseudo-1st order model was the best fit to explain the kinetic data across all the pH values tested. It showed the highest correlation values (R^2^) of 0.9885, 0.9958, and 0.9861 for pH values of 3, 6, and 10, respectively, as well as the lowest k values. Accounting for the assumptions of the pseudo-1st order kinetic model, adsorption occurred through diffusion through the interface; hence, the rate of adsorption was proportional to the number of available active sites on the BC surface [56,75]. For temperature dependence, the pseudo-1st order kinetic model fits the kinetic data better with R^2^ of 0.9981 and 0.9958 for temperatures 4 °C and 23 °C, respectively. However, at the elevated temperatures of 100 °C, the pseudo-2nd order model fits the kinetic model better with an R^2^ value of 0.9964. This indicated that at lower temperatures, the amount of available active sites on the biochar was the limiting step in the MB dye adsorption, while at higher temperatures, the rate-limiting step was the chemical adsorption [73]. The models were fitted to the experimental data and can be seen in Supplemental Information Figures S1 and S2 for pH dependence and temperature dependence, respectively.

#### 3.1.3. Nitrogen Sorption/BET Analysis Studies

The specific surface area analyses obtained via Brunauer–Emmett–Teller (BET) analysis are shown in Table 4. The BET surface area was 66.03 m^2^/g with a micropore volume of 0.04 cm^3^/g. The relatively low surface areas obtained could be attributed to low temperature for the slow pyrolysis during biochar formation as well as the blockage of pores by inorganic materials [76]. This is characteristic of biochar produced at temperatures ranging from 300 to 600 °C and made of a mixture of various woods [10,77,78].

### 3.2. Membrane Characterization

#### 3.2.1. Morphological Analysis via Scanning Electron Microscopy

**Figure 4:**
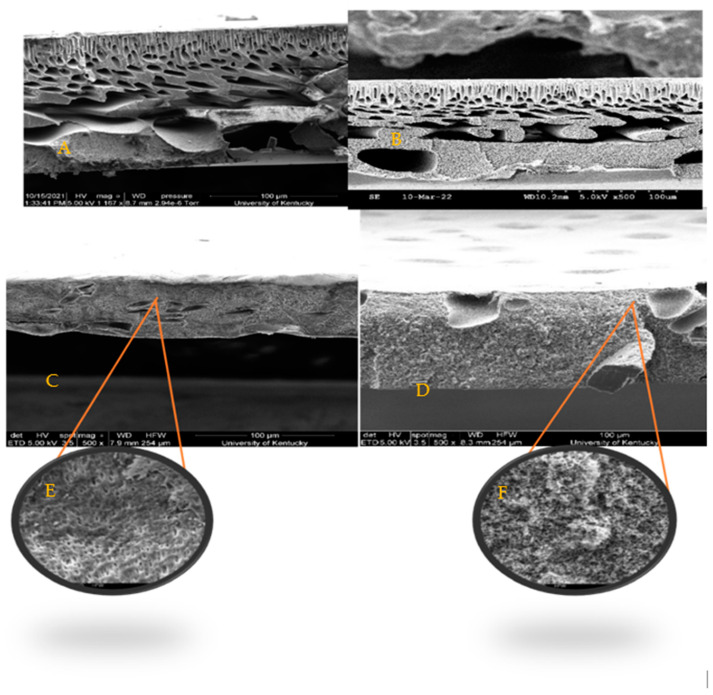
SEM images obtained at X500 showing the cross-sections of membranes (**A**) PSf-NMP, (**B**) BC-PSf-NMP, (**C**) BC-PSf-GVL, (**D**) BC-PSf-PC, (**E**) BC-PSf-GVL zoomed in, and (**F**) BC-PSf-PC zoomed in.

SEM images, Figure 4, show similar finger-like structures of both control membranes (PSf/NMP) in A as well as the membrane with biochar incorporated in it (BC-PSf/NMP) in B. Finger-like pore structures are often attributed to fast/instantaneous mixing and de-mixing of the solvent and the non-solvent [24]. The membranes synthesized from eco-friendly solvents GVL and PC in Figure 4C,D, respectively, presented fewer micro voids and more of a spongelike pore structure, which is consistent with those reported in the literature [24]. Zoomed in, the BC-PSf-GVL membranes exhibited more of an open-cell spongelike structure (E) while those of BC-PSf-PC showed an open interconnected spongelike structure (F). The sponge-like pore structure can be attributed to a slowed-down mixing and de-mixing process. The active layers of the biochar-based membranes were more visible as opposed to those of the control membranes, especially in the membranes made from the NMP solvent. This could be attributed to the high surface area and adsorbent quality of biochar [79]. Incorporating biochar into the PSf membrane matrix can slow down the rate of de-mixing of the solvent and non-solvent, allowing for the thicker active layer to form. This is because it behaves as a pore former capable of suppressing the micro voids with the membranes and affecting mass transport across the membrane surface [24].

#### 3.2.2. Elemental Analysis via XPS Analysis

**Figure 5:**
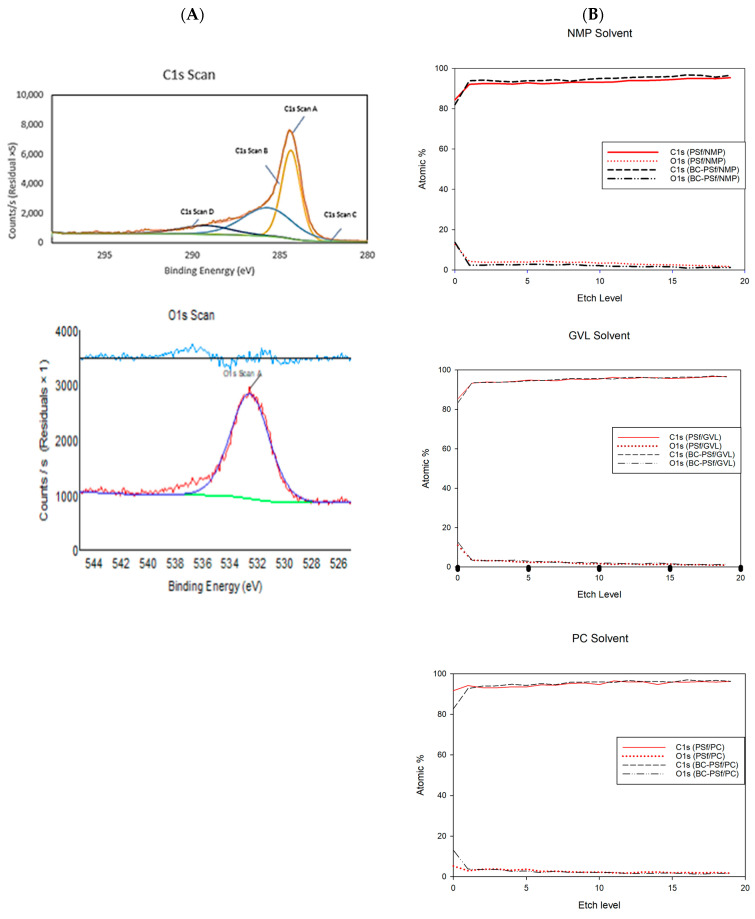
(**A**) fitted XPS analysis for elements C1s and O1s of the solid biochar. (**B**) XPS depth profiles of C1s and O1s found in membranes made with NMP, GVL, and PC solvents.

The elemental analysis of biochar showed that the biochar used consisted of only carbon and oxygen at 82.19% and 17.81%, respectively, as seen in Figure 5A. The C1s content consisted mainly of three types of peaks with 47.33, 40.23, and 11.83 atomic %, which corresponds to the C-C, C-O-C, and O-C=O type of bonding [80,81,82]. When incorporated into the membranes, XPS analysis could not show significant differences in the membranes with biochar incorporated in them as compared to those without biochar, as observed in Table 5. This can be attributed to the low percentage of biochar incorporated in the membrane dope solutions. An overall increase in the binding energy of the specified elements was observed when biochar was introduced into the membranes.

From the individual element fitted spectra in Figures S3 and S4, across all membranes, the C-C bonds contributed the highest to the C1s content; this was followed by the C-O-C bond and finally O-C=O bond. This was especially pronounced in the membranes without biochar incorporation made with NMP as a solvent. With the incorporation of biochar into the membrane matrix, the difference between the atomic weight percentages of the C-C and C-O-C bonding in the C1s spectra reduced significantly. For example, in the PSf-NMP membranes, much of the scan contributed to C-C bonding making up 78.72% of the C-content. Next, the C-O-C type of bonding contributed to 13.02% of the carbon content and the O-C=O type of bonding contributed to 5.97% of the carbon content. Comparing this to the BC-PSf-NMP membrane’s C1s spectra, the C-C content contributed to 53.72% of the carbon content while the C-O-C bonding contributed to 31.73% of the carbon content, and O-C=O contributed to 11.07% of the carbon content. For the membranes made from bio-derived solvents, the difference in the C1s scan’s bonding was not as pronounced.

For the O1s content, their spectra split from one peak to two peaks with the incorporation of biochar into the membranes. For example, in the O1s scan for the membranes made with GVL as the solvent, both membranes exhibited two peaks, which can be attributed to the S=O and S=C of PSf which should be at around 530 eV [81]. The peaks in the BC-PSf-GVL are more pronounced than those of PSf-GVL and more evenly distributed with the content of 58.32% and 41.68% for 531.96 and 533.55 eV, respectively, as opposed to those of the PSf-GVL, which had 66.45% and 33.55% for 532.49 and 534.04 eV, respectively.

To further understand how the biochar was incorporated into the membrane matrix, an XPS depth profile was carried out. For the membranes made with NMP as a solvent, in both the PSf-NMP and BC-PSf-NMP membranes, there was an increase in the atomic percent of both the C1s and a reduction in O1s content as the membranes were etched. The increase and decrease in the C1s and O1s, respectively, was higher in the PSf-NMP membranes. This meant that as membranes were etched further, more carbon was found. This corresponded with the results provided in Table 5, where there was an overall increase in the C1s content as biochar was introduced into the matrix. The same trend was observed in the membranes made from GVL and PC as seen in Figure 5B.

#### 3.2.3. Structural Analysis via FTIR

During NIPS, a complete phase transition is realized when the solvent is removed from the polymer and replaced with water [83,84]. It is expected that the solvent should not affect the functional properties, such as contact angle measurements of the formed membranes. However, it was observed that the choice of solvent had a significant impact on the pore microstructure of the formed membrane. This is because the interaction between the solvent and the non-solvent determines the rate at which NIPS will take place. If both solvent and non-solvent have a high diffusion rate, instantaneous de-mixing occurs, which can lead to microvoids, as observed in the SEM images in Figure S6. However, when the diffusion rate differs or is low, delayed de-mixing takes place, which could potentially lead to a bicontinuous pore structure [83]. FTIR analysis indicated that the formed membranes contain similar functional groups, and the incorporation of biochar into the membrane matrix did not alter the functionality of the membranes, as seen in the FTIR spectra. This can be seen in Figure S5 where C-H-stretch, S=S=O both symmetric and unsymmetric, aromatic backbone, as well as the C=O functional groups were observed at wavelengths of 3100, 1151, 1297, 1490, and 1650 cm^−1^ [21], respectively. Since biochar is composed mostly of carbon and oxygen, and given the low amount of biochar incorporated, PSf was expected to be the biggest contributor to membrane functionality. There was a slight shift of the stretching frequencies of some functional groups to higher wavenumbers as well as the introduction of new wavenumbers. The spectra of the membranes synthesized from NMP solvent showed a peak at a wavelength of 1689 cm^−1^. However, that peak shifted down to wavenumber 1681 cm^−1^ in the spectra of membranes synthesized with the eco-friendly solvents PC and GVL, as well as an introduction of a small new peak at wavenumber 1739 cm^−1^. The shift in the frequencies as well as the introduction of the new peaks could be attributed to the formation of hydrogen bonds between the PSf, biochar, and the solvents as new OH-O=S=O-OH bonds were formed [85].

#### 3.2.4. Structural Analysis via Mechanical Strength Testing

**Figure 6:**
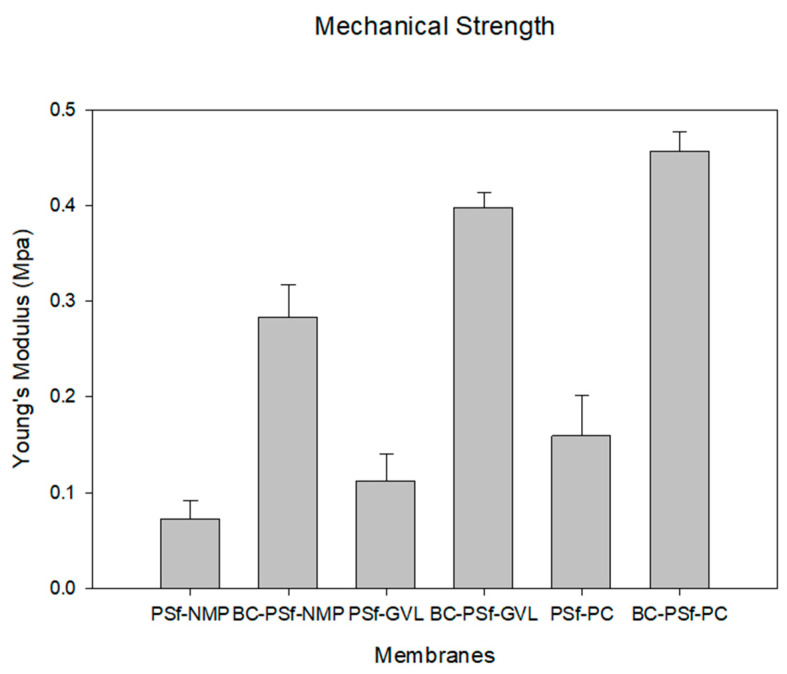
Graph showing the mechanical strength of the synthesized membranes as a function of Young’s modulus.

The ability of the synthesized membranes to resist elastic deformation can be seen via Young’s Modulus in Figure 6. Analysis of the mechanical properties of these membranes is essential in predicting the membranes’ life span and mechanical strength. With the introduction of biochar into the membrane matrix, there was a considerable increase in Young’s modulus irrespective of the solvent utilized. The BC-PSf-NMP membranes exhibited a 2.89 times increase in Young’s modulus as compared to the PSf-NMP membranes, the BC-PSf-GVL membranes exhibited a 2.53 times increase in Young’s modulus compared to the PSf-GVL membranes, and finally, the BC-PSf-PC membranes exhibited a 1.85 times increase in Young’s modulus as compared to the PSf-PC membranes. This indicated that the presence of biochar enhanced the mechanical strength of PSf, thereby potentially enhancing the lifespan of the membranes while maintaining their mechanical integrity. The mechanical strength analysis in Figure 6 shows that biochar-based membranes made from eco-friendly solvents exhibited stronger mechanical strength as opposed to those made with petroleum-derived solvent NMP. This could be attributed to the stronger interaction between the non-solvent and the eco-friendly green solvents during phase inversion during the NIPS process. From cross-sectional SEM images in Figure 4, the pores showed more of a spongelike anatomy, using the green solvents as opposed to the finger-like pores obtained using NMP as a solvent. The interconnected membrane matrix with fewer microvoids could have been attributed to the improved mechanical properties [86] of these membranes [87,88] for example, membranes with PC solvent exhibited the highest Young’s modulus.

#### 3.2.5. Functional Studies via Wettability Studies/Contact Angle Measurements

**Figure 7:**
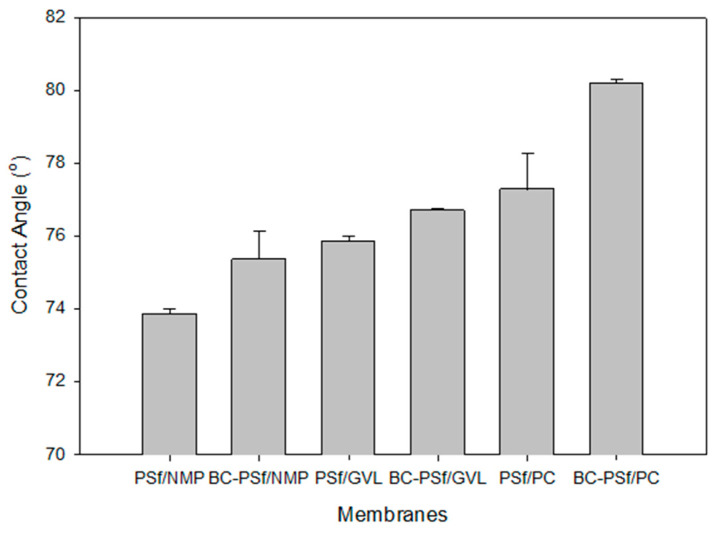
Graph showing the wettability of the synthesized membranes via contact angle measurements.

One of the most common ways to understand the wettability as well as the surface homogeneity [89] of a membrane is via contact angle measurements [89,90]. Due to the hydrophobic nature of most organic contaminants/foulants, the hydrophobicity of a membrane is an important indicator of the potential for fouling. The higher the contact angle, the more likely it will foul and vice versa [91]. Figure 7 shows the contact angle of the synthesized membranes. There was a slight increase in the membranes with biochar incorporated in them as opposed to those without. Furthermore, the membranes synthesized with eco-friendly solvents exhibited higher contact angle measurements, indicating that they were more hydrophobic and hence prone to fouling as opposed to those synthesized with petroleum-derived solvents. Since there is a direct relationship between hydrophobicity and mechanical strength [85], the results of the contact angle measurements complimented those of the mechanical strength measurements, as seen in Figure 6, where the green solvents not only had higher mechanical strength but also higher contact angle measurements. This was especially true for PC solvent, which exhibited the highest values for both parameters. The NIPS process indicates that solvent is washed out of the pores and should therefore not influence the wettability of the membranes. The contact angle measurements could be attributed to the pore structure or residual solvent bound on the surface of the membrane.

#### 3.2.6. Effect of Membrane Surface Charge via Zeta Potential Studies

One of the most reliable indicators of membrane surface charge is zeta potential. It gives predictive information on the membrane’s fouling capabilities [92]. Zeta potential is not directly measured but is rather the electrical resistance in pores of potassium chloride electrolyte at varying pH. The ratio of electrolyte conductivity inside the membrane pores to the bulk conductivity gives an idea of the isoelectric point concerning the pH [93]. Table 6 shows the zeta potential of the membrane surfaces of PSf-NMP, BC-PSf-NMP, PSf-GVL, BC-PSf-GVL, PSf-PC, and BC-PSf-PC at pH of 3, 6, and 10. At a pH of 3, all the membranes demonstrated negative zeta potential values. The membranes with biochar incorporated into them were found to have significantly higher zeta potential values than those without biochar. Furthermore, membranes synthesized with eco-friendly solvents GVL and PC had a bigger impact on the zeta potential value when biochar was incorporated compared to those synthesized from petroleum-derived solvent NMP. This agreed with the results obtained from the mechanical strength and contact angle measurements in Figure 6 and Figure 7, respectively. The zeta potential results showed that apart from the BC-PSf-NMP membranes, the incorporation of biochar into the membranes significantly increased the zeta potential and hence made the membranes more negatively charged. Furthermore, the use of eco-friendly solvents resulted in membranes with higher zeta potential and higher negative charge. A pH of 6 gave the most negative zeta potential across all membranes. For the membranes without biochar in them, the negative zeta potential can be attributed to the adsorption of anions of electrolyte (Cl^−^) and hydroxyl ions (OH^−^) onto the surfaces from the HCL and NaOH solution used for pH adjusting. For the membranes with biochar incorporated in them, on top of the OH^−^ and Cl^−^ from the electrolyte solution, further -COOH groups from the biochar could be contributors to the surface charge formation. The XPS analysis further supports this, where especially in the eco-friendly solvents, the O1s peaks split into two peaks and the ratio between the C-C and O-C=O peaks in the C1s peak was closer showing the introduction of biochar into the membrane matrix (Figures S4 and S5). It could be deduced that the transport properties of the synthesized biochar membranes were more influenced by the change in the pH of the feed, since zeta potential is caused by the adsorption of anions onto the membrane surfaces [92].

#### 3.2.7. Membrane Structural Analysis via TGA Studies

TGA was carried out to identify the effect of biochar and eco-friendly solvents on thermal stability as a function of the weight (%) of PSf membranes. From Figure S7, all the membranes exhibited their first weight loss at 130 °C. This could be attributed to the solvent residuals and moisture present within the membranes [94]. The highest weight loss due to solvent residuals was exhibited by the membranes synthesized from PC solvent. This could explain the increased contact angle measurements obtained for those membranes. Because biochar is a byproduct of lignocellulosic materials, its thermal stability should be lower than that of the PSf polymer; hence, the membranes with biochar incorporated in them should show more than one step of decomposition, as the second step should be indicative of the decomposition of the hemicellulose and lignin left in the biochar [95]. However, incorporation of the biochar did not show such an effect. This could be attributed to the low percentage of biochar incorporated. Looking at the solvents, it was evident in the membranes made from eco-friendly solvents, especially GVL, that there was more than one decomposition step, as it is a byproduct of cellulosic biomass. This showed that in the membranes synthesized, the solvent utilized had a higher effect on thermal stability than the incorporation of biochar in its matrix.

#### 3.2.8. Stability Studies via Viscosity Measurements

**Figure 8:**
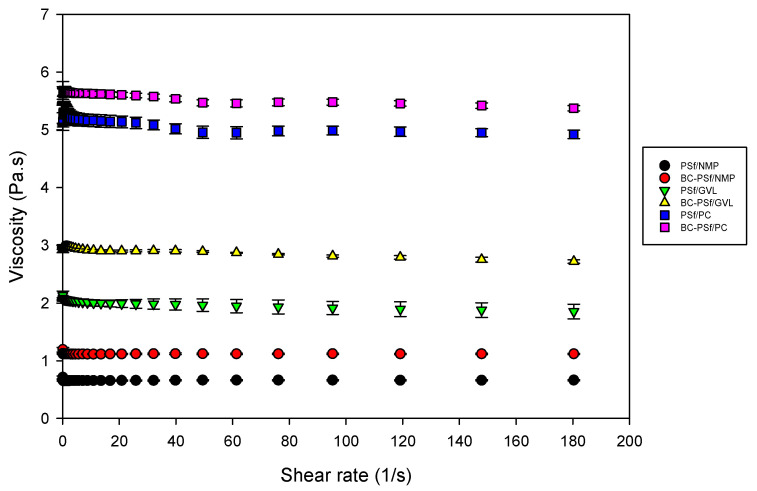
Graph showing the viscosity of dope solutions of membrane composites synthesized from various solvents.

The viscosity was measured, as shown in Figure 8. Incorporation of biochar into the PSf membrane matrix increased the dynamic viscosity of the dope solutions with the biggest increase being in the membranes synthesized from GVL solvent. This indicated that there would be delayed de-mixing of solvent and non-solvent in the membranes with biochar incorporated in them. Furthermore, membranes synthesized from eco-friendly solvents GVL and PC exhibited significantly higher viscosity compared to those synthesized from petroleum-derived solvent NMP; thus, the membranes from eco-friendly membranes should exhibit delayed de-mixing, resulting in more active layers [96], as observed in the SEM images in Figure 4. Membranes synthesized from PC as a solvent had the highest viscosity, which correlates to their high contact angle (increased hydrophobicity) in Figure 7 and higher mechanical strength in Figure 6.

### 3.3. Adsorptive Properties of the Biochar-Based Membranes

**Figure 9:**
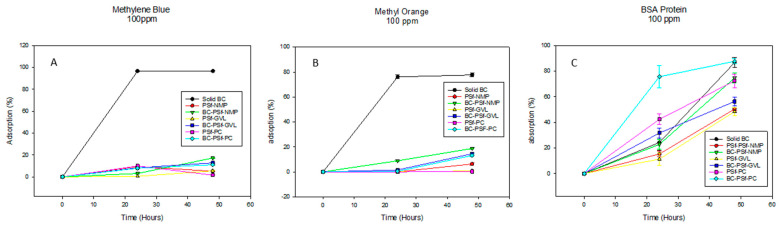
Graphs showing the adsorption percentage of methylene blue dye, methyl orange dye, and BSA protein at 100 ppm in (**A**), (**B**), and (**C**), respectively. For better visualization of the data, graphs were not started at the 0.0 point.

The adsorptive properties of the synthesized membranes were first evaluated by letting the membranes soak in 100 ppm solutions of both positively and negatively charged dyes methylene blue and methyl orange, as shown in Figure 9A,B, respectively, as well as 100 ppm of BSA solution, shown in Figure 9C. The membranes without biochar incorporated into them exhibited increased adsorption properties of methylene blue dye after 24 h. However, after 48 h, there was diminished adsorption as compared to 24 h, which indicated that there was desorption of the dye from the membrane surface over time. However, there was a continued increase in the adsorption of the dye with increased time. This showed that biochar increased the adsorptive properties of PSf membranes for methylene blue dye. For the negatively charged dye methyl orange (Figure 9B), there was increased dye adsorption across all membranes regardless of the solvent used or the incorporation of biochar, with BC-PSf-NMP showcasing the highest adsorption percentage. This was repeated utilizing BSA protein (Figure 9C) to confirm the results from a larger negatively charged contaminant and similar results were observed. Relating to the zeta potential in Table 6, since there was a negative charge across the membranes at a pH of 6, this indicated that charge repulsion of the negatively charged BSA protein was also taking place.

**Figure 10:**
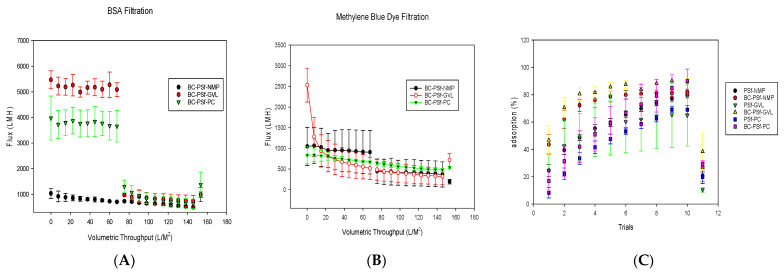
Graphs showing the flux analysis of 100 ppm BSA filtration in (**A**) as well as flux analysis and dye adsorption of methylene blue dye at 10 ppm in (**B**,**C**). For better visualization of the data, the graphs were not started at the 0.0 point.

Flux analysis was carried out for the biochar–PSf membranes in the various solvents, using BSA protein at 100 ppm and methylene blue dye at 10 ppm as feed water, under dead-end filtration at a constant pressure of 4.1 bar, as seen in Figure 10A,B, respectively. Compared to PSf/NMP membranes, the biochar membranes displayed either similar levels of flux or significantly higher levels depending on the solvent used. Since BSA protein has been utilized extensively throughout the literature to study the flux performance of PSf membranes, it was utilized alongside the methylene blue dye in the filtration studies as a control. This gave information on the repulsive nature of the membranes, as BSA should be big enough to filter through an ultrafiltration membrane. BSA protein (Figure 10A) filtration through membranes of BC-PSf/NMP showed the initial flux values at an average of 800 ± 7 LMH for pre-compaction. There was a slight reduction in the flux during BSA filtration with the initial flux at 727 ± 26 LMH and the final being at 492 ± 33 LMH; however, during backflow, the flux was recovered back to pre-compaction levels, indicating that any fouling accumulation on the membranes was reversible, along with the reusability and recyclability of the synthesized membranes.

On the other hand, the membranes made using eco-friendly solvents exhibited an increased flux performance both during pre-compaction and 100 ppm BSA filtration. This was more evident for the BC-PSf-PC membranes. Though BC-PSf-NMP membranes had significantly higher pre-compaction flux as compared to BC-PSf-NMP membranes, the flux values of the BSA filtration were almost double those of the membranes with NMP as solvent. These results were consistent with those obtained by Lu et al., where the membranes synthesized from eco-friendly membranes exhibited increased permeability(3.5%) as well as rejection of BSA protein (53.2%) [97]. The contact angle of these membranes was also observed to be higher than those of the membranes with NMP solvent, as shown in Figure 7. Increases in contact angle are directly proportional to increases in hydrophobicity; therefore, PSf/PolarClean membranes were found to be more hydrophobic. Since BSA protein is hydrophobic in nature [21], there should be a higher tendency for the membrane to foul due to the repulsive forces between the hydrophobic membrane surface and the hydrophobic protein. This explains the instantaneous fouling that took place when the protein solution was filtered through the membranes. This is because membrane-foulant hydrophobic adsorption mainly affects initial fouling or irreversible fouling, where the foulant directly contacts the membrane surface [98]. The spongelike pore structure experienced by the membranes made from eco-friendly solvents showed a high interconnectivity, which can reduce the hydrophobic adsorption (Figure 4). This is because the walls of the pores have a limited contactable area for the adsorption; thus, the foulants are not stable under applied pressure [98]. The high interconnectivity of the porous substructure of these membranes provides reduced flux values. This provides better mechanical properties, as defined by mechanical strength (Figure 6) compared to the fingerlike structure [99]. For this, the eco-friendly solvent-based membranes should have a higher flux due to their sponge-like pore structure formed.

As can be observed in Figure 4C,D, though both membranes made using GVL and PC solvents showed spongelike pore structures, membranes made with GVL displayed more of a closed-cell structure while those made with PC had an open-pore interconnected structure. This also played a role in how the foulant BSA protein interacted with the membrane surfaces. The flux of the membranes with open-pore interconnected structures has been shown to exhibit higher flexibility hence higher mechanical strength with increased permeability/flux compared to closed-cell pore structures [100].

**Figure 11:**
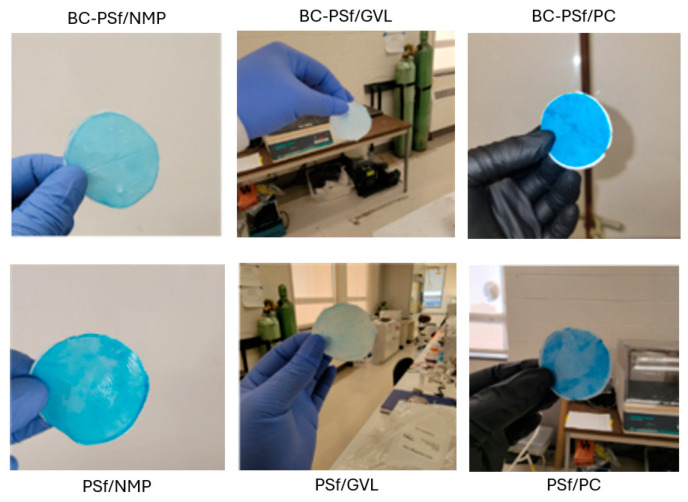
Images of the various membranes showing the membrane after methylene blue dye filtration.

Organic dyes have been extensively utilized in fields such as the textile industry, leather tanning industry, paper production, food technology [101], etc. This could potentially lead to their leaching into natural waters and their removal is necessary for improved water quality. Figure 10B,C show both the flux performance and the rejection/adsorption percentage of methylene blue dye at 10 ppm. The concentration was reduced from 100 to 10 ppm to avoid the instantaneous fouling associated with the high dye concentration that would have otherwise filled up all the active sites for binding during dye filtration. Similar to BSA filtration, BC-PSf-GVL membranes had the highest initial pre-compaction values; however, there was no considerable difference in the flux performance of the membranes during BSA filtration. Backflow showed that the membranes synthesized from eco-friendly solvents had slightly higher flux values but not back to pre-compaction levels. This showed that methylene blue dye was irreversibly adsorbed onto the membranes and could not be removed by physical means. BC-PSf-GVL membranes exhibited the highest percentage adsorption of the dye in Figure 10C whereby 80% of the dye had adsorbed onto the membrane surface, followed by BC-PSf-NMP membranes and lastly BC-PSf-PC membranes (trial 6). The results can be physically observed on the filtration membranes via Figure 11.

### 3.4. Membrane Stability via Leaching Studies

#### 3.4.1. Soaking Method (Leaching Study)

**Figure 12:**
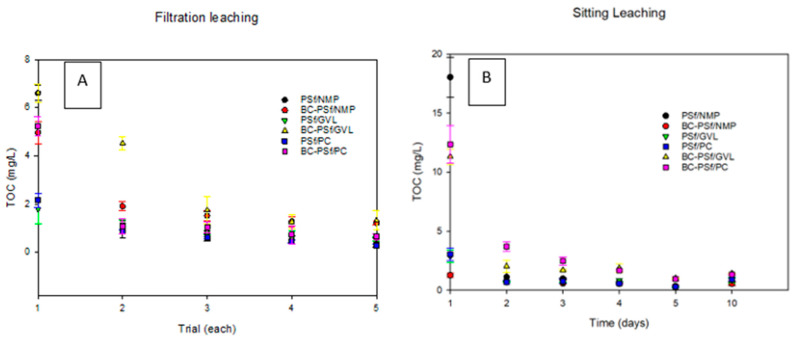
Graphs showing the leaching of the synthesized membranes via the sitting method in (**A**) and the filtration method in (**B**).

Figure 12A shows the TOC data of the samples of all the synthesized membranes from the sitting/soak (Figure 12A) leaching test. In all the membranes, much of the leaching was observed on the 1st day of the test, and by the 2nd day of the test, leaching had stabilized/ceased. Graphs for each membrane composite can be found in Figure S8. For the membranes synthesized from NMP solvent, the membranes with biochar incorporated into them in S8B showed minimal leaching as indicated by the low TOC values compared to those without biochar in S8A. For the membranes synthesized with bio-derived solvents, membranes with biochar exhibited significantly increased TOC values (Figure S8D,F for GVL and PC solvents, respectively), as opposed to those without biochar (Figure S8C,E for GVL and PC, respectively). This indicated that biochar was leaching out of the membranes synthesized from eco-friendly solvents faster than those from petroleum-derived membranes.

#### 3.4.2. Filtration Method (Leaching Study)

Figure 12B shows the TOC data of the samples of all the synthesized membranes from the filtration leaching test. Just like in the soaking test, in all the membranes, much of the leaching was observed on the first trial of the test, and by the fourth trial of the test, leaching had stabilized. Graphs for each membrane composite can be found in Figure S9. Membranes with biochar (Figure S9B,D,F for NMP, GVL, and PC solvents, respectively) exhibited significantly increased TOC values as opposed to those without biochar (Figure S9A,C,E for NMP, GVL, and PC solvents, respectively). This indicated that biochar was leaching out of the synthesized membranes, apart from the membranes synthesized with NMP as a solvent. Here, though the initial TOC value for the BC-PSf-NMP membranes (Figure S9B) was higher than that of the PSf-NMP (Figure S9A), the average TOC was higher for the rest of the test days. Looking at the effect of solvent on the leaching of the membranes, it was observed that the membranes synthesized from bio-derived solvents GVL and PC had lower TOC values, indicating less leaching in those membranes as compared to those synthesized from petroleum-derived solvent NMP. Both the soaking/sitting and the filtration tests indicated similar results for the biochar leaching from membranes.

### 3.5. LCA Results

#### 3.5.1. Unit Environmental Impacts of Materials

**Figure 13:**
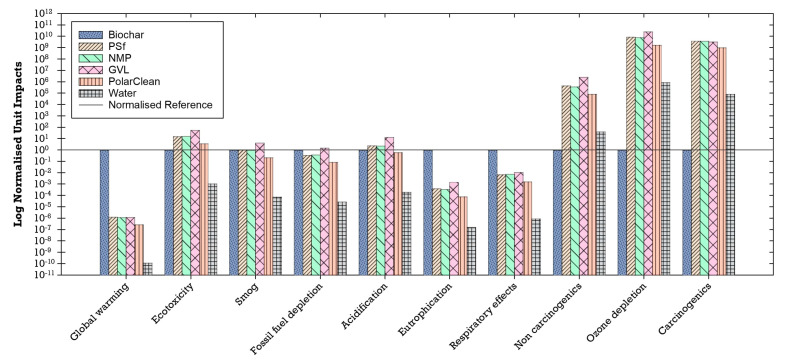
Environmental unit impacts of the materials used normalized to biochar (log scale).

The unit impacts of the materials used in the production of the flat-sheet membranes (i.e., impacts per 1 kg of material) were compared to each other to understand their individual environmental impacts. The unit environmental impacts normalized to biochar showed that biochar has higher unit impacts for global warming, eutrophication, and respiratory effects than the other materials (Figure 13). Global warming potential is likely attributed to gases such as methane emitted into the atmosphere during the biochar production process as modeled in SimaPro [41,102]. Non-methane volatile organic compounds, carbon monoxide, nitrogen oxides, and particulate matter (PM_10_)—which are airborne particles with a diameter of less than or equal to 10 µm—are primary air pollutants produced during the biochar production process and thus may be linked to the observed respiratory effects that can lead to chronic and acute respiratory diseases [41,103]. Similarly, nitrogen from the nitrogen oxides emitted during combustion in the biochar process may be associated with high levels of eutrophication [41,104]. Furthermore, life cycle inventory processes for PSf, NMP, GVL, PolarClean, and water (which includes processes for treatment and distribution) have higher unit environmental impacts in impact categories of non-carcinogenic, ozone depletion, and carcinogenic compared to biochar (Figure 13).

#### 3.5.2. Comparison of Global Environmental Impacts of Membrane Configurations

**Figure 14:**
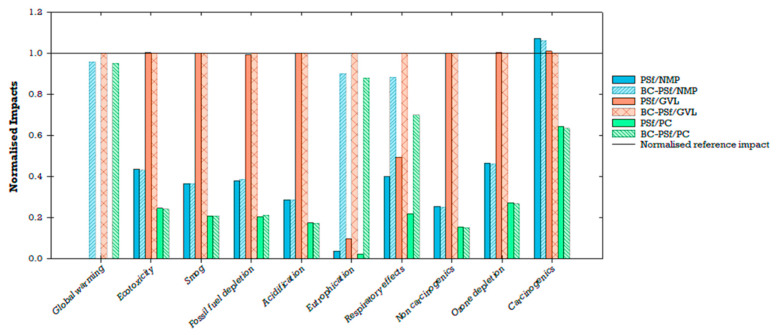
Normalized environmental impact results (per 1000 m^2^ of BC/GVL/PSf).

Six membrane configurations were compared based on their global environmental impacts (PSf/NMP, BC-PSf/NMP, PSf/GVL, BC-PSf/GVL, PSf/PC, and BC-PSf/PC; Figure 14). Results were normalized to 1000 m^2^ (functional unit) of the biochar–polysulfone–gamma-valerolactone (BC-PSf/GVL) membrane for comparative analysis. This specific membrane was selected for normalization due to its consistently higher environmental impacts across multiple categories when compared to other membranes. Notably, PSf/NMP and BC-PSf/NMP employed a petroleum-derived solvent (NMP), whereas PSf/GVL, BC-PSf/GVL, PSf/PC, and BC-PSf/PC utilized eco-friendly solvents (GVL and Rhodiasolv^®^ PolarClean, PC) to enhance the performance of PSf or BC/PSf membranes. PSf, a prominent petroleum-based synthetic polymer in membrane fabrication, is favored in membrane fabrication compared to other conventional polymers like cellulose acetate for its high thermal resistance, chemical resistance, and mechanical strength [27]. On the other hand, biochar, a black carbon-rich product, has demonstrated notable sorption and removal capacities for various water pollutants [13,16,17,18].

Membranes employing GVL as a green solvent exhibited higher environmental impacts compared to those using the traditional solvent (NMP) and, to a lesser extent, PolarClean. Additionally, the incorporation of biochar in the membranes significantly increased global warming impacts, eutrophication, and respiratory effects, while exerting a smaller influence on carcinogenic impacts. Membrane configurations with biochar (BC/PSf/NMP, BC/PSf/GVL, and BC/PSf/PC) exhibited higher impacts compared to those without biochar (PSf/NMP, PSf/GVL, and PSf/PC). The biochar membranes when compared to non-biochar membranes showed slightly lower impacts on the carcinogenic category, while variations in other impact categories (e.g., ecotoxicity, smog, fossil fuel depletion, acidification, non-carcinogenic, and ozone depletion) were much smaller. Specifically, BC/PSf/NMP had a 4%, 1%, and 1% lower contribution to global warming, eutrophication, and respiratory effects, respectively, while BC/PSf/PC had 5%, 1%, and 3% lower contributions compared to BC/PSf/GVL at the same scale. The non-biochar membranes made a much smaller contribution to global warming.

In contrast, membrane configurations incorporating biochar performed better in the carcinogenic impact category. Particularly, PSf/NMP and BC-PSf/NMP demonstrated a 7% and 6% higher impact on carcinogenic compared to the reference BC-PSf/GVL membrane. PSf/GVL showed a 1% increase in carcinogenic impacts compared to BC-PSf/GVL. PSf/PC and BC-PSf/PC showcased a 36% and 37% reduction in carcinogenic impacts compared to the reference membrane, with PSf/PC marginally higher than BC-PSf/PC. From the findings, it can be concluded that the decision on which membrane performs better environmentally depends on the specific environmental impact category and priorities for application. If the focus is on minimizing contributions to global warming, eutrophication, and respiratory effects, the non-biochar membranes (especially PSf/PC) perform relatively better. However, when considering uncertainty, carcinogenic impacts for the membranes incorporating biochar (especially BC-PSf/PC) demonstrated similar global environmental impacts compared to the reference membrane (Figure 14).

**Figure 15:**
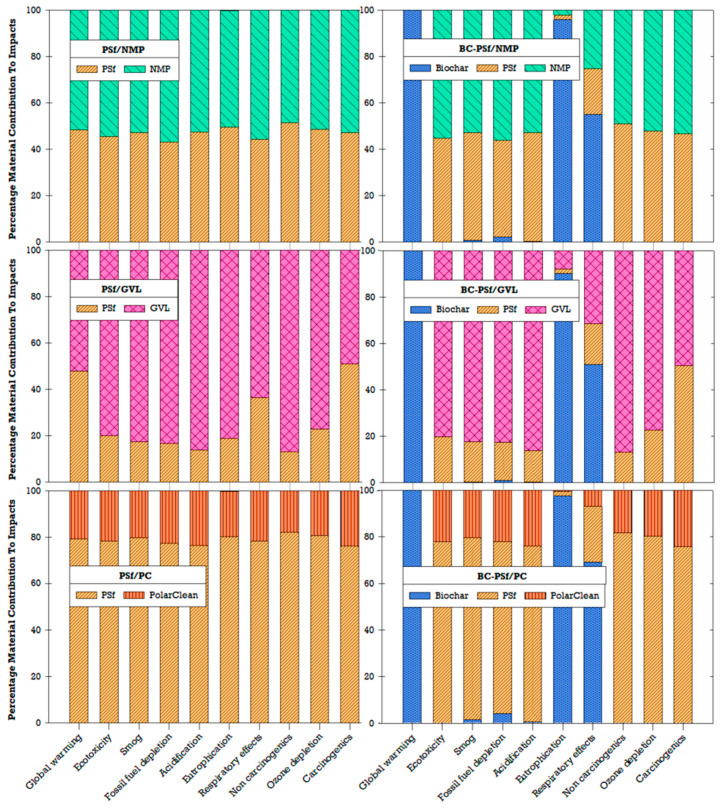
Percentage of material contributions to environmental impacts.

Due to concerns related to environmental and human health impacts, previous studies have recommended the replacement of traditional or petroleum-derived solvents (e.g., NMP) with green solvents (e.g., GVL, PC) [27,32,35]. Similarly, incorporating biochar in the production of membranes has been found to enhance the effectiveness of biochar as a sorbent, which could be beneficial for water treatment applications [18]. By understanding the environmental implications of the choice of solvent and incorporating biochar, better material choices and decisions can be made to further improve the sustainability of membrane fabrication. LCA results showed that the choice of solvent affects the environmental impact of fabricated membranes (Figure 15). Among the three solvents compared for the non-biochar membranes, GVL had the highest impact contribution, followed by NMP, and lastly PolarClean (PC). In the PSf/PC membrane configuration, the environmental impacts were driven by the polymer (PSf) rather than the solvent (PC). The addition of biochar contributed significantly to global warming, eutrophication, and ozone depletion compared to other impact categories (Figure 15). Ultimately, tradeoffs between performance and global environmental impacts will need to be navigated depending on the context and stakeholder goals.

#### 3.5.3. Uncertainty Analysis of Environmental Impacts and Sensitivity to Membrane Fabrication Materials

**Figure 16:**
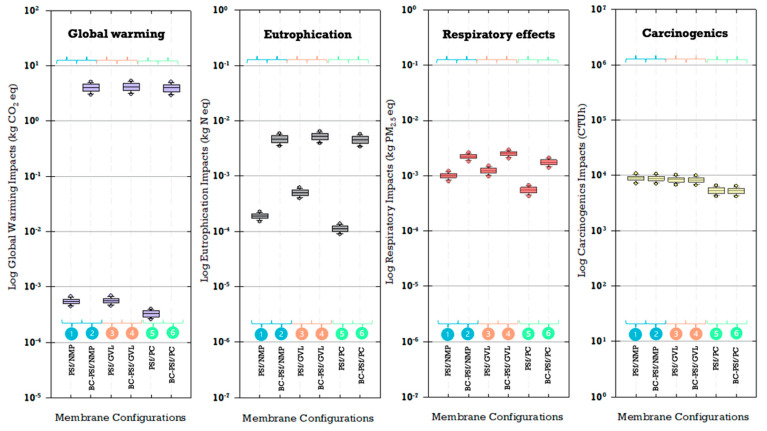
Uncertainty of selected impact categories.

Variability in the amount of material during membrane fabrication may cause uncertainty in the quantified environmental impacts. Uncertainty analysis results revealed a substantial difference in global warming and eutrophication impacts between biochar and non-biochar membranes (Figure 16). Biochar membranes exhibited impacts higher by four orders of magnitude for global warming and one order of magnitude for eutrophication. This underscores the influence of biochar addition in membrane fabrication for these specific impact categories. The differences in magnitude between biochar and non-biochar membranes were much less for the respiratory effects impact category. On the other hand, carcinogenic impacts were impacted by the choice of solvent more than the inclusion of biochar. Notably, membranes synthesized with PolarClean resulted in median carcinogenic impacts that were the lowest for PolarClean and highest for NMP, though there was a large overlap in these ranges when considering uncertainty. For impact categories of global warming, eutrophication, and respiratory effects, non-biochar membranes with PolarClean consistently had the lowest median environmental impacts. Interestingly, biochar membranes demonstrated lower median carcinogenic impacts when synthesized with PolarClean.

**Figure 17:**
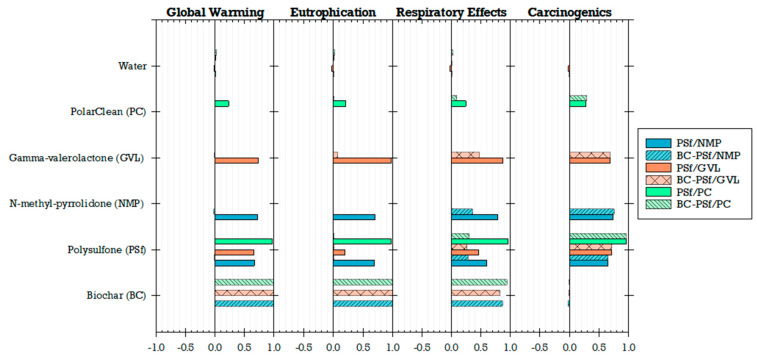
Sensitivity of impacts to membrane materials.

Sensitivity analysis results further highlighted the influence of biochar addition on global warming, eutrophication, and respiratory effects (Figure 17). Notably, the traditional polymer, particularly PSf, played a crucial role in shaping the environmental impacts of the PSf/PC membrane (Spearman’s correlations ranged from 0.95 to 0.97 across all impact categories). This correlation trend extended to biochar PSf/PC membranes for the impact categories other than global warming, eutrophication, and respiratory effects (correlations of 0.0005, 0.02, and 0.3, respectively). Similar patterns emerged in other membranes synthesized from both biochar and non-biochar using solvents like NMP and GVL. A comparison of solvent choices revealed that environmental impacts were least sensitive to PolarClean (Spearman’s correlations ranging from 0.004 to 0.3 across all impact categories), suggesting its potential as a green solvent for sustainable membrane production. However, this study revealed a tradeoff between functional performance and environmental impacts if biochar is to be used. Regarding functionality, integrating biochar into the membrane matrix not only enhanced the mechanical properties but also improved performance metrics related to flux and adsorption percentages. This enhancement was particularly notable in membranes synthesized using GVL solvent, where the incorporation of biochar led to a significant 40% increase in MB dye adsorption. On the other hand, biochar membranes synthesized with GVL exhibited elevated environmental impacts in the categories of global warming, eutrophication, and respiratory effects. The sensitivity analysis indicated that biochar was the predominant factor driving impacts in these impacts (Spearman’s correlations ranging from 0.83 to 0.99 across all impact categories), compared to GVL (Spearman’s correlations ranging from 0.02 to 0.5 across all impact categories).

## 4. Conclusions

This work evaluated the effect of incorporating biochar into the PSf membrane matrix and compared the use of petroleum-derived solvent NMP to the use of bio-derived solvents GVL and PC. This was analyzed by investigating the role of pore morphology, contact angle measurements, mechanical strength tests, and adsorption of both positively and negatively charged dyes. It was also found that the incorporation of biochar into the membrane matrix not only improved the mechanical properties of the membranes but also improved the performance in terms of flux and adsorption percentages of the synthesized membranes. Furthermore, membranes synthesized from eco-friendly solvents GVL and PC exhibited improved performance compared to those synthesized from petroleum-derived solvent NMP. Morphologically, membranes synthesized from NMP solvent (petroleum-derived solvent) exhibited finger-like pore structures with larger microvoids. On the other hand, membranes synthesized from eco-friendly solvents GVL and PC exhibited a spongelike pore structure, which was attributed to an increase in their wettability. The improved performance was especially evident in membranes synthesized from GVL solvent where there was a 40% increase in MB dye adsorption when biochar was incorporated. Leaching studies carried out under both filtration and soaking methods suggested increased membrane leaching with the incorporation of biochar into the membrane matrix. There was reduced leaching in the membranes synthesized from NMP as compared to those synthesized from the eco-friendly solvents GVL and PC.

This study further presented a comparative life cycle assessment (LCA) of six distinct membrane synthesis configurations (PSf/NMP, BC-PSf/NMP, PSf/GVL, BC-PSf/GVL, PSf/PC, and BC-PSf/PC), each having a standardized surface area of 1000 m^2^ as the functional unit. The use of biochar, NMP, GVL, and PC in these membrane matrices was investigated for associated global environmental impacts, accompanied by uncertainty and sensitivity analyses. Normalization of environmental impacts to the BC-PSf/GVL membrane revealed an increase in global warming impacts, eutrophication, and respiratory impacts due to biochar addition, with minimal influence on carcinogenic impacts. Importantly, environmental impacts were found to be highly sensitive to biochar addition (Spearman’s correlation coefficient >0.8 across all impact categories). It is important to note that biochar could be produced using processes and technologies that differ from what was modeled in this study; therefore, environmental impacts for biochar-based membranes could be improved. This would be especially true if biochar were produced with other feedstocks or along with additional co-products, thus providing beneficial offsets to the environment. In conclusion, this study shows that biochar as well as eco-friendly solvents (e.g., PolarClean) offer benefits and tradeoffs for functional performance and global environmental impacts.

## Figures and Tables

**Table 1:** Percentage volume composition of materials for each membrane configuration for the dope solution required to produce 1 m^2^ membrane (linearly scaled to the functional unit of 1000 m^2^).

		Membrane Configurations					
	Units	PSf/NMP	BC/PSf/NMP	PSf/GVL	BC/PSf/GVL	PSf/PC	BC/PSf/PC
Materials	kg/m^2^	0.457	0.457	0.461	0.461	0.451	0.451
Biochar	%	0	1	0	1	0	1
PSf	%	46	45	47	46	45	44
NMP	%	54	54	0	0	0	0
GVL	%	0	0	53	53	0	0
PolarClean	%	0	0	0	0	55	55
Water	kg/m^2^	2.46	2.46	2.46	2.46	2.46	2.46

**Table 2:** The parameters for the Langmuir, Freundlich, and Sips isotherms for MB dye adsorption at pHs 3, 6, and 10.

Isotherms	Langmuir			Freundlich			Sips		
Parameters	q_m_ (mg/g)	k_D_ (L/mg)	R^2^	n	K (L/mg)	R^2^	n	K (L/mg)	R^2^
pH 3	121910.0	6.4 × 10^−5^	0.7495	1.08	1014.3	0.9377	0.45	0.022	0.8760
pH 6	1764145.7	9.2 × 10^−4^	0.9534	0.80	790.7	0.8615	1.10	44.027	0.9483
pH 10	321765.3	5.2 × 10^−3^	0.8202	1.10	1104.4	0.8238	3.46	0.003	0.7091

**Table 3:** The parameters for both Pseudo-1st and Pseudo-2nd order kinetic adsorption models under varying pHs and temperatures.

**Kinetic Models**	**Pseudo-1st order**			**Pseudo-2nd order**	
Parameters	K_1_	R^2^		K_2_	R^2^
pH 3	0.013	0.9885		1.22 × 10^11^	0.9777
pH 6	0.005	0.9958		2.80 × 10^12^	0.7571
pH 10	0.002	0.9861		4.09 × 10^11^	0.6350
**Kinetic Models**	**Pseudo-1st order**			**Pseudo-2nd order**	
Parameters	K_1_		R^2^	K_2_	R^2^
RT (23 °C)	0.005		0.9958	2.80 × 10^12^	0.7571
Cold (4 °C)	0.0141		0.9981	3.18 × 10^8^	0.8066
warm (100 °C)	0.0158		0.9899	1.16 × 10^13^	0.9964

**Table 4:** Surface area results obtained via BET analysis.

BET Surface Analysis	
BET Surface Area (m^2^/g)	66.03
Langmuir Surface Area (m^2^/g)	84.84
Micropore Volume (cm^3^/g)	0.04
External Surface Area (m^2^/g)	2.09
Single Point Surface Area (m^2^/g)	67.31
Micropore Area (m^2^/g)	68.12

**Table 5:** The elemental composition of the solid biochar as well as membranes synthesized from NMP, GVL, and PC as a function of their atomic weight percent and peak binding energy.

**PSf/NMP**			**BC-PSf/NMP**		
**Name**	**Peak BE**	**Atomic %**	**Name**	**Peak BE**	**Atomic %**
C1s	284.38	80.8	C1s	286.86	80.13
O1s	532.01	15.71	O1s	534.64	14.45
S2p	167.89	1.87	S2p	169.99	3.31
Ca2p	347.11	1.29	Ca2p	347.1	0
N1s	399.3	0.32	N1s	401.62	1.48
**PSf/GVL**			**BC-PSf/GVL**		
**Name**	**Peak BE**	**Atomic %**	**Name**	**Peak BE**	**Atomic %**
C1s	285.44	80.98	C1s	285.45	80.89
O1s	533.34	14.22	O1s	533.23	15.63
S2p	168.9	3.09	S2p	168.92	2.96
Ca2p	348.33	1.29	Ca2p	348.34	0
N1s	400.41	0.41	N1s	407.08	0.52
**PSf/PC**			**BC-PSf/PC**		
**Name**	**Peak BE**	**Atomic %**	**Name**	**Peak BE**	**Atomic %**
C1s	285.13	81.58	C1s	285.12	83.21
O1s	532.85	14.24	O1s	532.83	13.32
S2p	168.37	3.2	S2p	168.31	3.45
Ca2p	348.1	0	Ca2p1	348.08	0
N1s	400.08	0.3	N1s	399.02	0

**Table 6:** Zeta potential for the synthesized membranes.

	pH 3			pH 6			pH 10	
Membranes	Zeta (mV)	S/D (mV)	Membranes	Zeta (mV)	S/D (mV)	Membranes	Zeta (mV)	S/D (mV)
PSf-NMP	−10.75	1.1	PSf-NMP	−30.09	2.1	PSf-NMP	−29.68	1.7
BC-PSf-NMP	−5.27	2.1	BC-PSf-NMP	−41.50	4.2	BC-PSf-NMP	−30.59	4.6
PSf-GVL	−4.22	4.9	PSf-GVL	−54.16	1.8	PSf-GVL	−52.60	1.2
BC-PSf-GVL	−44.12	7.5	BC-PSf-GVL	−80.38	10.4	BC-PSf-GVL	−66.20	3.5
PSf-PC	−14.26	7.4	PSf-PC	−21.81	9.3	PSf-PC	−17.77	1.1
BC-PSf-PC	−47.28	11.0	BC-PSf-PC	−83.97	12.6	BC-PSf-PC	−71.38	6.5

## Data Availability

The original contributions presented in the study are included in the Article/Supplementary Materials, further inquiries can be directed to the corresponding author.

## Supplementary Materials

The following supporting information can be downloaded at: https://www.mdpi.com/article/10.3390/membranes14070153/s1, The Supplementary Materials contain additional graphs of the kinetic models used for the adsorptive studies of MB dye on biochar, details on the life cycle inventory, the mathematical computational structure of the MATLAB code for life cycle assessment, and the GitHub link to the code repository [40,41,42].
