# Supplementary material for: Performance and Environmental Assessment of Biochar-Based Membranes Synthesized from Traditional and Eco-Friendly Solvents
Source: Membranes (Basel). 2024 Jul 11;14(7):153. doi: 10.3390/membranes14070153 (PMC11279014; doi:10.3390/membranes14070153)
Supplement: Supplementary file 1 [file membranes-14-00153-s001.zip › membranes-3064574-supplementary.pdf]

membranes

MDPI

Supplementary Materials

Performance and Environmental Assessment of Biochar-Based Membranes Synthesized from Traditional and Eco-Friendly Solvents

Abelline Fionah [1], Isaac Oluk [2], Laura Brady [3], Diana M. Byrne [2] and Isabel C. Escobar [3,*]

1 Department of Chemistry, University of Kentucky, Lexington, KY 40506, USA; akfionah1@uky.edu
2 Department of Civil Engineering, University of Kentucky, Lexington, KY 40506, USA; isaacoluk@uky.edu (I.O.); dianabyrne@uky.edu (D.M.B.)
3 Department of Chemical and Materials Engineering, University of Kentucky, Lexington, KY 40506, USA; lbrady12@vols.utk.edu
* Correspondence: isabel.escobar@uky.edu

SI1: Membrane fractional components

Table S1 in the supplemental section shows the specified conditions for each membrane formed. It further shows the conditions for the synthesis of the various membrane composites used in the study such as the percent total polymer, the percentage of biochar added to the dope solution, the temperature at which the dope solution was stirred, and finally the time it took for total dissolution of the polymer in each solvent.

Table S1. Fractional components as well as the conditions used in dope solution formulation for the membrane synthesis (*Total polymer constitutes biochar and PSf for membranes with biochar).

	PSf/NMP	BC-PSf/NMP	PSf/GVL	BC-PSf/GVL	PSf/PC	BC-PSf/PC
Polymers/additive	PSf/-	PSf/BC	PSf/-	PSf/BC	PSf/-	PSf/BC
Solvent	NMP	NMP	GVL	GVL	PC	PC
% total Polymer*	17	17	17	17	17	17
% BC additive	–	2	–	2	–	2
% solvent	83	83	83	83	83	83
Dope solution temp (°C)	23	80	80	80	80	80
Dissolution time (days)	2	6	2	8	2	8

SI2: Additional Graphs for Kinetic Models

Figures S1 and S2 in the supplemental sections show the graphs of the kinetic models used for the adsorptive studies of MB dye on biochar. Figure S1 shows the pH dependence of the dye absorbed onto the substrate biochar while Figure S2 shows the temperature dependence of the dye solution absorbance onto the substrate biochar. The figures show the adsorption capacities of the biochar at given times in A, D, and G for pH's 3, 6, and 10 respectively. Then they show the pseudo 1st order and pseudo 2nd order model fittings at each respective pH.

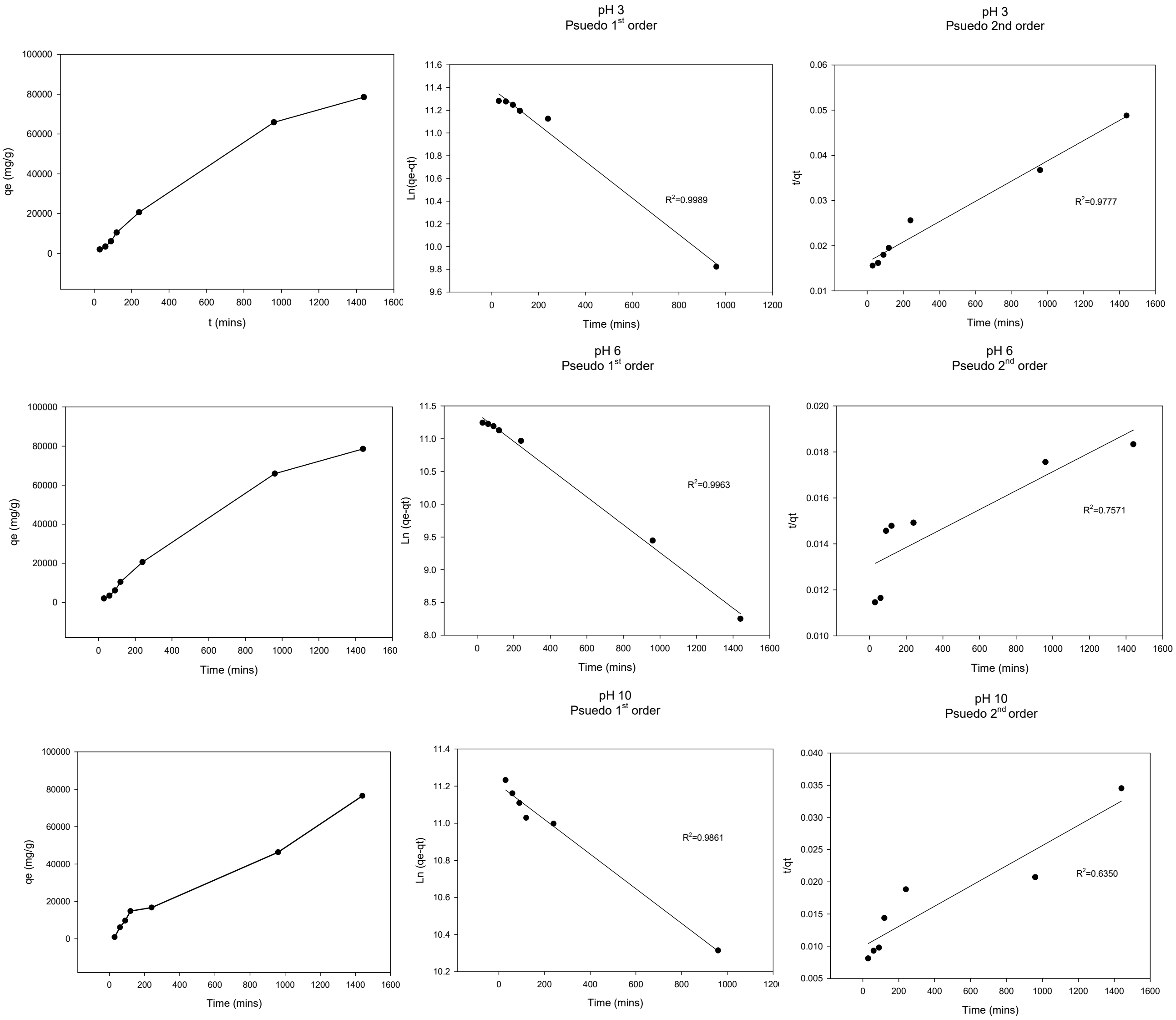

Figure S1. Graphs of Kinetic models' pseudo-1st and pseudo-2nd order fitted to experimental data for MB adsorption onto BC at pHs 3, 6, and 10.

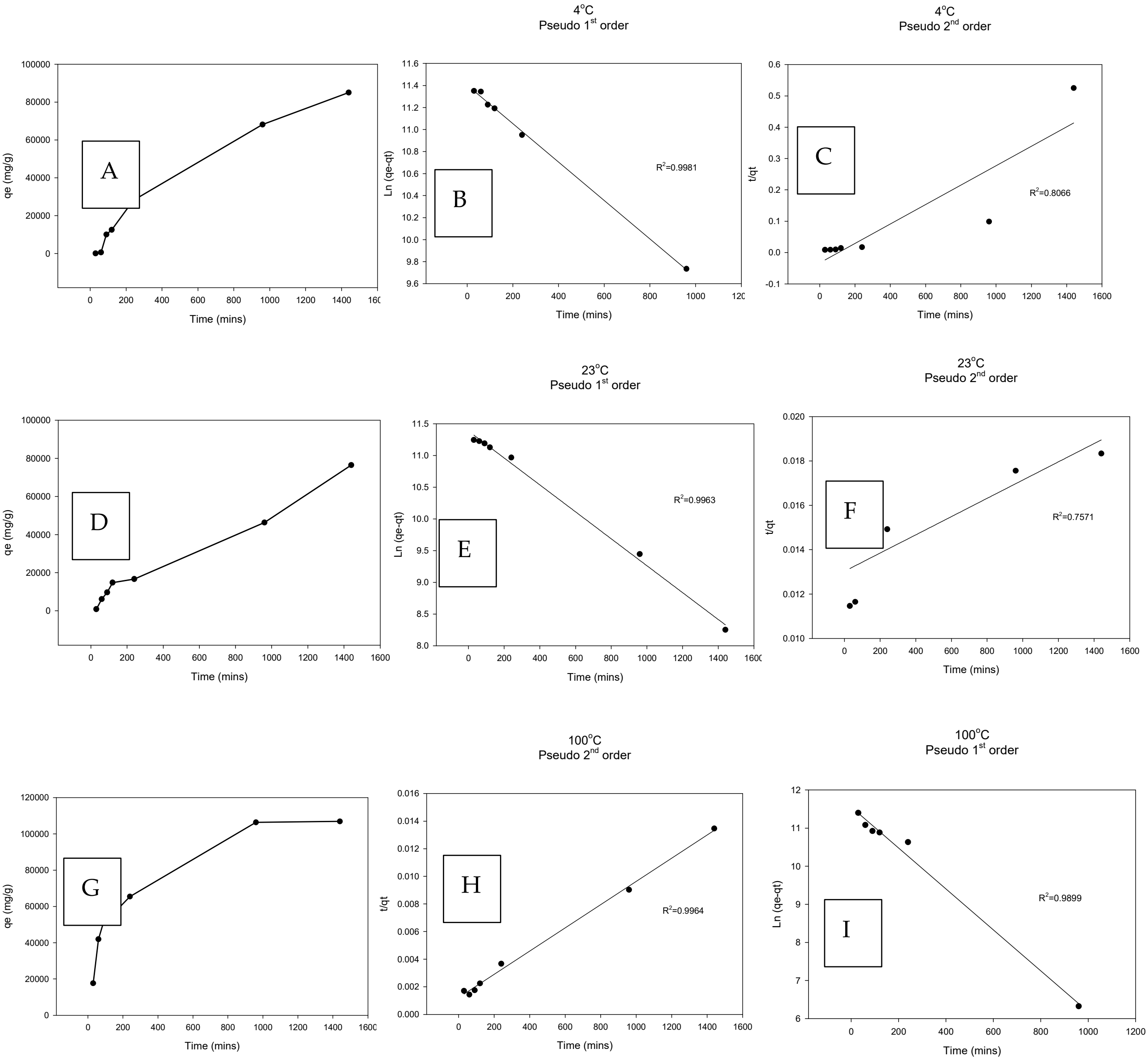

Figure S2. Graphs of Kinetic models' pseudo-1st and pseudo-2nd order fitted to experimental data for MB adsorption onto BC at pHs Temperatures 4°C, 23°C, and 100°C.

SI3: functional and elemental analysis

Figures S2-S5 show the elemental analysis of the synthesized membranes containing the biochar additive. The XPS spectra and table results in Figures S3, S4, and S5 show the xps data fitted for membranes synthesized from each of the solvents utilized NPM, GVL, and PC in Figures S3, S4, and S5 respectively. The spectra of the selected scans C1s and O1s are on the Left while the tables on the right show the fitted data extracted from XPS analysis. Figure S6 shows the functional analysis of the membranes analyzed via Ft-IR. TGA analysis can be found in Figure S7. Membrane leaching test results from both the soaking and filtration tests can be found in Figures S8 and S9 respectively.

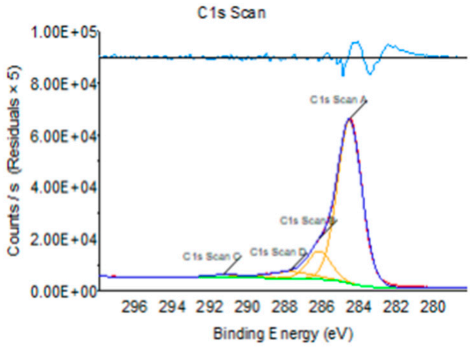

PSf/NMP Carbon		
Name	Peak BE	Atomic %
C1s Scan A	284.52	78.72
C1s Scan B	286.14	13.02
C1s Scan C	291.27	2.29
C1s Scan D	287.72	5.97

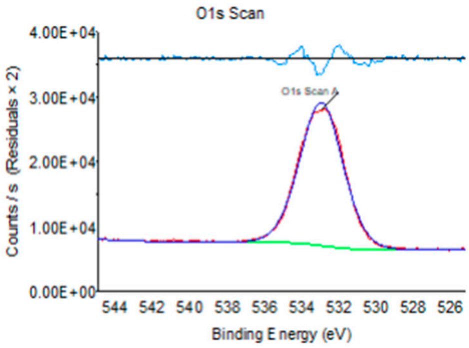

PSf/NMP Oxygen		
Name	Peak BE	Atomic %
O1s Scan A	532.95	100

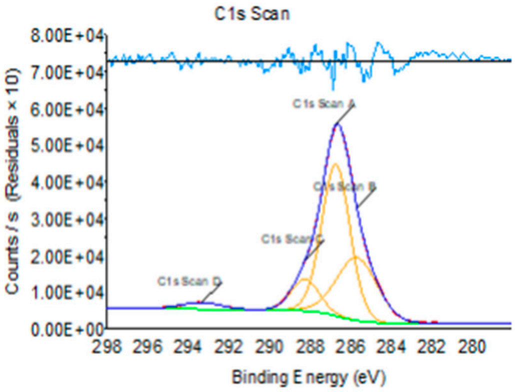

BC-PSf-NMP Carbon		
Name	Peak BE	Atomic %
C1s Scan A	286.76	53.72
C1s Scan B	285.72	31.73
C1s Scan C	288.26	11.07
C1s Scan D	293.35	3.48

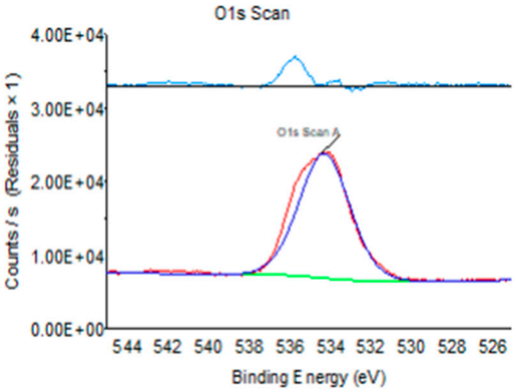

BC-PSf-NMP Oxygen		
Name	Peak BE	Atomic %
O1s Scan A	534.47	100

Figure S3. XPS spectra with fitting for selected elements C1s and O1s of PSF/NMP and BC-PSf-NMP membranes.

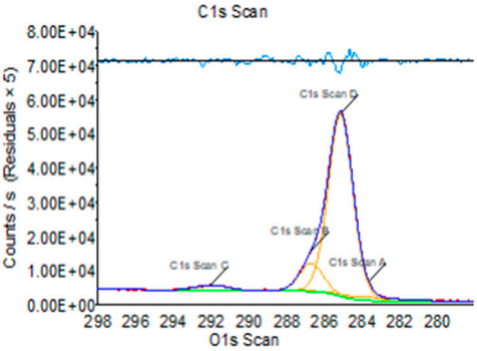

PSf-GVL Carbon		
Name	Peak BE	Atomic %
C1s Scan A	283.67	3.23
C1s Scan B	286.7	12.12
C1s Scan C	292.02	3.31
C1s Scan D	285.16	81.33

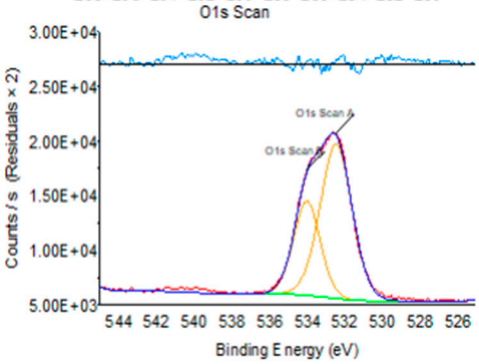

PSf-GVL Oxygen		
Name	Peak BE	Atomic %
O1s Scan A	532.49	66.45
O1s Scan B	534.04	33.55

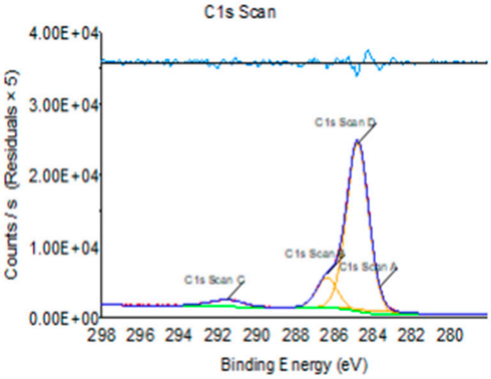

BC-PSf-GVL Carbon		
Name	Peak BE	Atomic %
C1s Scan A	283.68	3.49
C1s Scan B	286.39	13.22
C1s Scan C	291.56	4.78
C1s Scan D	284.81	78.51

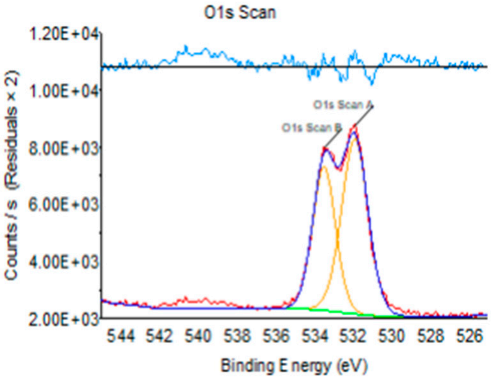

BC-PSf-GVL Oxygen		
Name	Peak BE	Atomic %
O1s Scan A	531.96	58.32
O1s Scan B	533.55	41.68

Figure S4. XPS spectra with fitting for selected elements C1s and O1s of PSF/GVL and BC-PSf-GVL membranes.

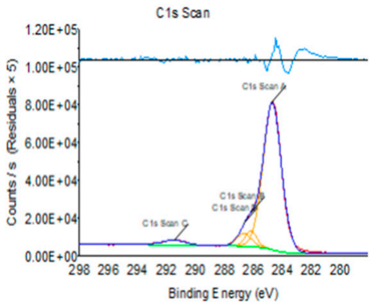

PSf-PC Carbon		
Name	Peak BE	Atomic %
C1s Scan A	284.72	84.18
C1s Scan B	286.18	5.73
C1s Scan C	291.47	4.21
C1s Scan D	286.66	5.89

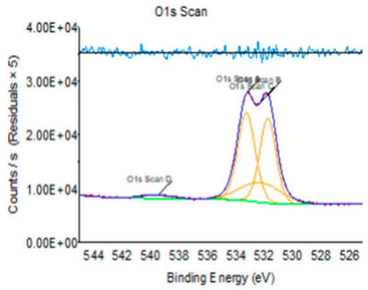

PSf-PC Oxygen		
Name	Peak BE	Atomic %
O1s Scan A	533.28	39.94
O1s Scan B	531.77	35.97
O1s Scan C	532.29	21
O1s Scan D	539.44	3.09

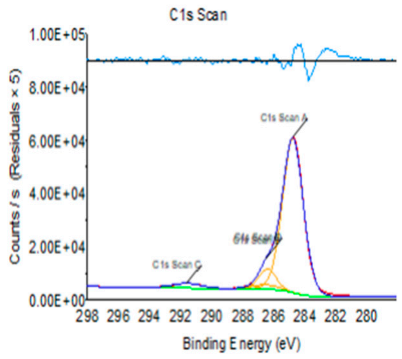

BC-PSf-PC Carbon		
Name	Peak BE	Atomic %
C1s Scan A	284.78	84.52
C1s Scan B	286.52	2.74
C1s Scan C	291.61	3.59
C1s Scan D	286.41	9.14

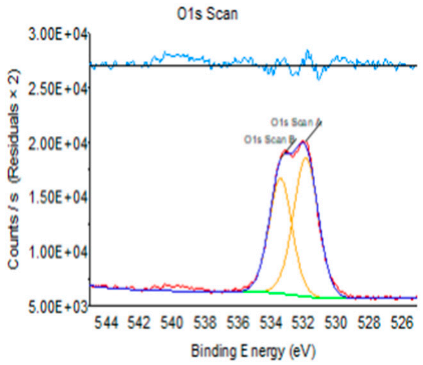

BC-PSf-PC Oxygen		
Name	Peak BE	Atomic %
O1s Scan A	531.88	56.39
O1s Scan B	533.43	43.61

Figure S5. XPS spectra with fitting for selected elements C1s and O1s of PSF/PC and BC-PSf-PC membranes.

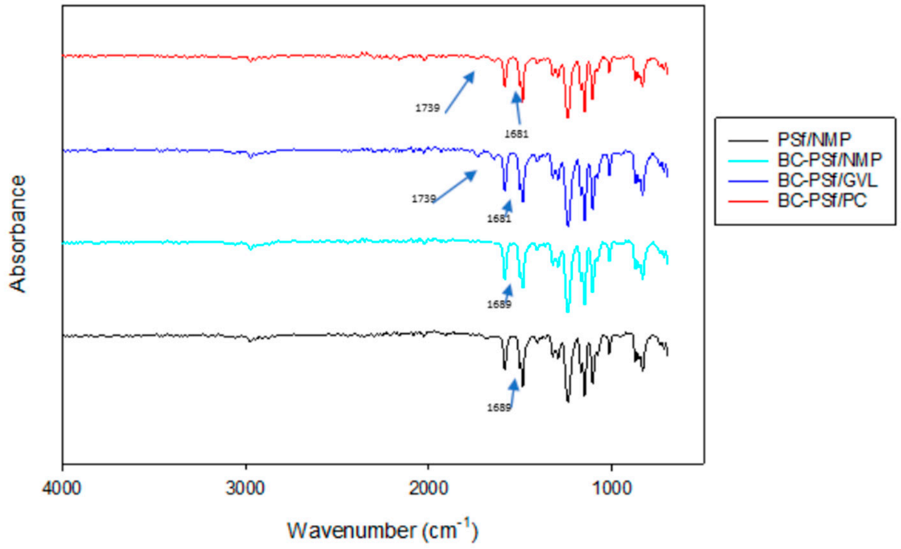

Figure S6. FTIR spectra of PSf-NMP, BC-PSf-NMP, BC-PSf-GVL, and BC-PSf-PC membranes.

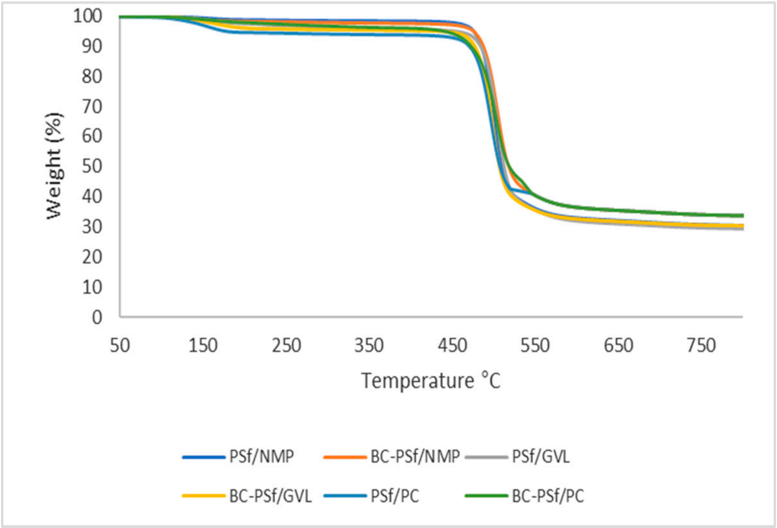

Figure S7. TGA curves of membranes made from various solvents.

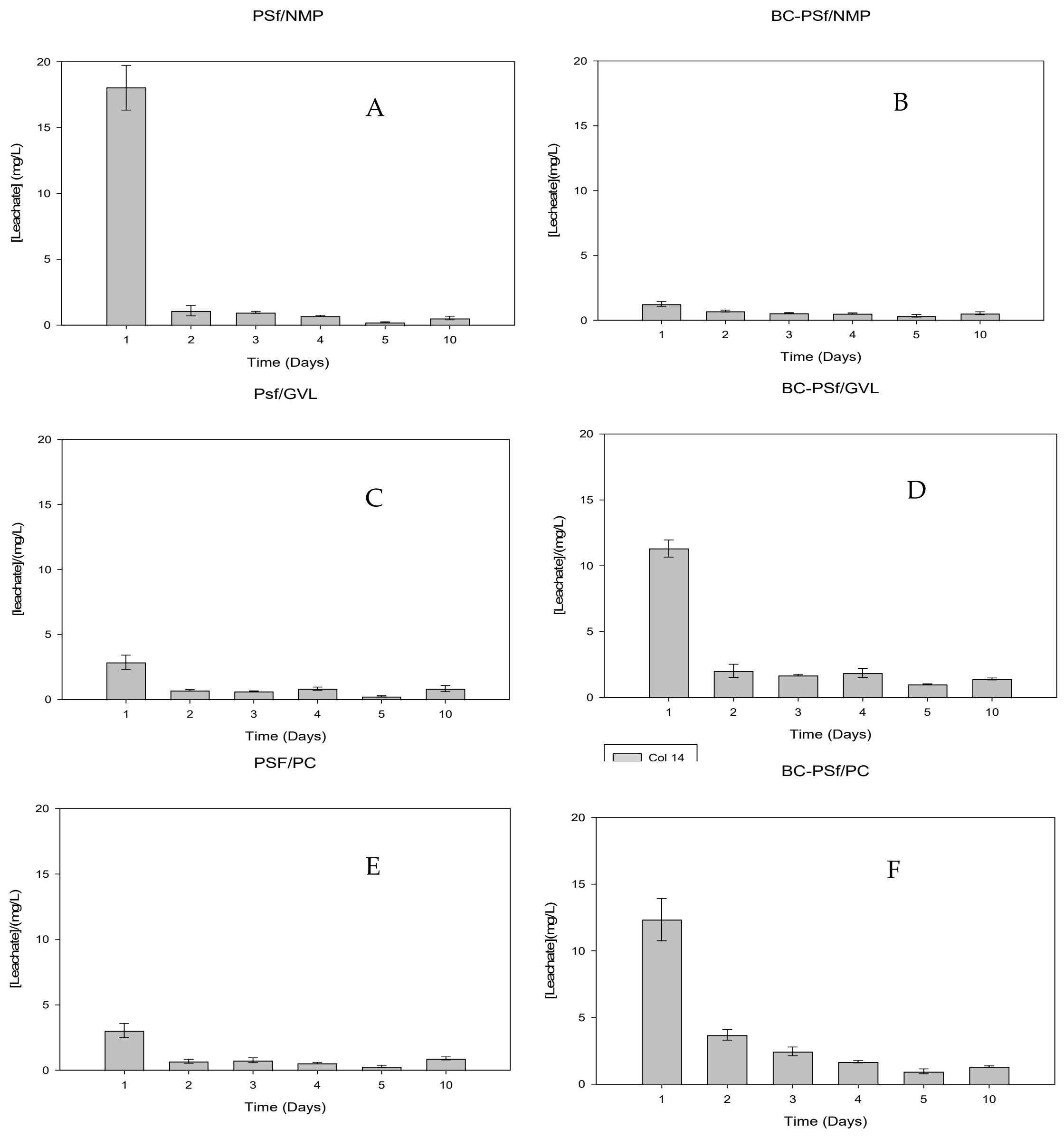

Figure S8. Graphs showing leaching of Biochar particles after sitting for specified periods of time for membranes of PSF/NMP in A, BC-PSf/NMP in B, PSf/GVL in C, BC-PSf/GVL in D, PSf/PC in E, and BC-PSf/PC in F

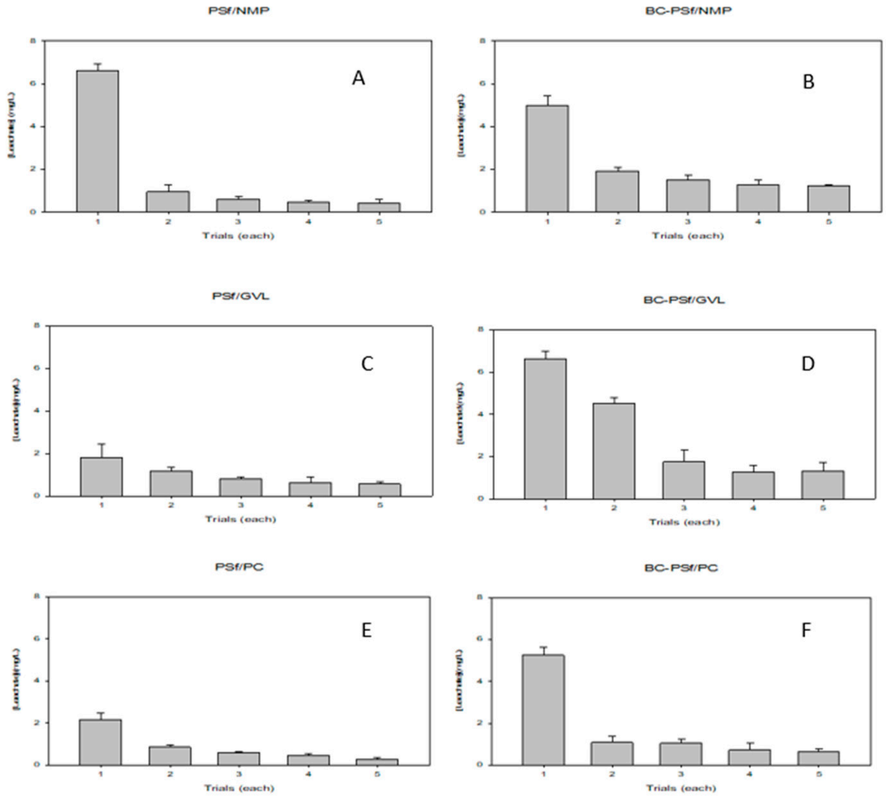

Figure S9. graphs showing leaching of Biochar particles after sitting for specified periods of time for membranes of PSF/NMP in A, BC-PSf/NMP in B, PSf/GVL in C, BC-PSf/GVL in D, PSf/PC in E, and BC-PSf/PC in F

SI4: Life Cycle Inventory

Table S2 provides the inventory for the laboratory-scale membrane fabrication process. Tables S3 to S8 below show the details of the inputs and outputs of all the life cycle inventory materials and processes based on literature and the Ecoinvent 3.5 database in SimaPro.

Table S2. Inventory for laboratory scale materials required to produce 1 m^2 of flat sheet membrane.

Materials	Units	PSf/NMP	BC-PSf/NMP	PSf/GVL	BC-PSf/GVL	PSf/PC	BC-PSf/PC
Biochar – BC	kg/m^2	0	0.004128	0	0.0043012	0	0.004096
Polysulfone – PSf	kg/m^2	0.21133	0.2072	0.2151	0.2107588	0.205	0.200724
N-methyl-pyrrolidone – NMP	kg/m^2	0.24603	0.2460327	0	0	0	0
Gamma-valerolactone – GVL	kg/m^2	0	0	0.246	0.2460327	0	0
Rhodiasolv® PolarClean – PC	kg/m^2	0	0	0	0	0.246	0.246033
Water	kg/m^2	2.46	2.46	2.46	2.46	2.46	2.46
Temperature conditions	^{o}C	25.0	80.0	80.0	80.0	80.0	80.0
Time (8-10 days)	days	2.0	9.0	9.0	9.0	9.0	9.0

Table S3. Inventory for producing biochar powder produced by slow pyrolysis of wood logs using a kiln-based technology. Inventory data for biochar powder was sourced from studies conducted by Smebye et al (2017) and Shaheen et al (2022).

Inputs			Outputs		
Nature	*Amount*	*Unit*	*Product*	*Amount*	*Unit*
Wood, feedstock[1]	5.932	kg	Biochar	1	kg
Wood, dry matter[2]	0.41	kg			
Technosphere	*Amount*	*Unit*			
Metalworking steel[3]	2.51E-4	kg			
Clay brick[3]	0.1	kg			
Electricity[4]	0.093	kWh			
			Emission to air[5]	*Amount*	*Unit*
			Carbon monoxide	0.15	kg
			Nitrogen oxides	1.7E-3	kg
			Methane	3.5E-2	kg
			PM_{10} SPM	7.69E-2	kg
			NMVOC	6.87E-2	kg

[1] High-voltage electricity is required for biochar sieving, washing, and crushing into biochar based on actual operation data [40].

[2]Forest residues (pulp logs) for slow pyrolysis into biochar. The feedstock is waste that otherwise would have degraded to biogenic CO_2, and a biochar yield of 36% is assumed [41]

[3] Start-up wood. In a retort kiln, start-up wood is burned for heat to create syngases before the pyrolysis process begins. The collection of wood is assumed to result in deforestation (assuming an above-ground biomass density of 29 kg/m^2 in a tropical forest), which then converts the forest to 50% arable and 50% shrubland, respectively [41].

[4] Average for product manufacturing of the kiln. A kiln consists of steel (100 kg) and bricks (4000 kg) which is normalized by 40 000 kg biochar, assuming a life expectancy for the kiln of 4 years and a biochar production of 10 000 kg per year [41]. All materials are from global averages [41].

[5] Emissions data from literature [41].

Table S4. Inventory for polysulfone, PSf based on Ecoinvent 3.5 database. All materials are from global averages unless specified. Applicable Ecoinvent process: Polysulfone {GLO}| polysulfone production, for membrane filtration production | APOS, U.

Inputs			Outputs		
Nature	*Amount*	*Unit*	*Product*	*Amount*	*Unit*
Oxygen	0.404	kg	Polysulfone	1	kg
Water, cooling	0.024	m^3			
Water	0.012	m^3			
Technosphere	*Amount*	*Unit*			
2,4-dichlorophenol	0.932	kg			
Benzene	0.999	kg			
Bisphenol A, powder	0.360	kg			
Chemical factory, organics	3.999E-10	kg			
Electricity, medium voltage	0.485	kWh			
Heat from steam {RER}	0.330	MJ			
Heat from steam {RoW}	1.669	MJ			

Emission to air	*Amount*	*Unit*
Phenol	0.000719996223455025	kg
Phenol, 2,4-dichloro-	0.00186381369987058	kg
Water/m³	0.0117599383164321	kg
Technosphere	*Amount*	*Unit*
Wastewater {CH}	0.000819386411916196	m³

{RER} and {RoW} are regional codes for Europe and Rest of World, respectively.

Table S5. Inventory for Gamma-valerolactone, GVL [42].

**Inputs**			**Outputs**		
Nature	*Amount*	*Unit*	*Product*	*Amount*	*Unit*
Textile, kenaf {GLO}	4.601	kg	Gamma-valerolactone	1	kg
Sulfuric acid {RoW}	0.046	kg			
Packing, lime product {GLO}	0.035	kg	Avoided products		
Water {RoW}	1.872	kg	Electricity	-2.164	kWh
Hydrogen, liquid {RoW}|	0.016	kg			
			Technosphere	*Amount*	*Unit*
			Waste gypsum {RoW}	0.081	kg
			Wood ash mixture {RoW}	0.286	kg

Table S6. Inventory for N-methyl-2-pyrrolidone, NMP. Applicable Ecoinvent process: N-methyl-2-pyrrolidone {GLO}| market for | APOS, U.

**Inputs**			**Outputs**		
Nature	*Amount*	*Unit*	*Product*	*Amount*	*Unit*
N-methyl-2-pyrrolidone {RER}	0.174	kg	N-methyl-2-pyrrolidone	1	kg
N-methyl-2-pyrrolidone {RoW}	0.826	kg			
Transport, freight train {GLO}	0.3091	tkm			
Transport, freight, inland waterways {GLO}	0.0246	tkm			
Transport, freight, lorry {GLO}	0.2088	tkm			
Transport, freight, sea {GLO}	0.599	tkm			

Table S7. Inventory for Rhodiasolv® PolarClean, PC.

**Inputs**			**Outputs**		
Technosphere	*Amount*	*Unit*	*Product*	*Amount*	*Unit*
Isobutanol {RoW}	0.201	kg	Rhodiasolv® PolarClean	1	kg
Dimethylamine {RoW}	0.0712	kg			
Acetic anhydride {GLO}	0.2705	kg			
Electricity, high voltage {GLO}	0.1	kg			
			Technosphere	*Amount*	*Unit*
			Wastewater {GLO}	0.5	m³

Table S8. Inventory for water. Applicable Ecoinvent process: Tap water {GLO}| market group for | APOS, U.

Inputs			Outputs		
Technosphere	*Amount*	*Unit*	*Product*	*Amount*	*Unit*
Tap water {CA-QC}	0.00112384533891088	kg	Tap water	1	kg
Tap water {RER}	0.400209800300613	kg			
Tap water {RoW}	0.598666354360476	kg			

Scheme 5. Computational structure of the LCA in MATLAB.

Mathematically, material amounts (kg /m²) represented by a 6 by 6 matrix (M) were linearly scaled to the functional unit by multiplying all inventory data by a demand scaling factor (d = 1000 m²) to obtain a life cycle inventory matrix (L) needed to produce different flat sheet membrane configurations (Equation S1).

$$L = d \times \begin{pmatrix} M_{1,1} & \cdots & M_{1,j} \\ \vdots & \ddots & \vdots \\ M_{i,1} & \cdots & M_{i,j} \end{pmatrix}$$

$$Therefore, L = \begin{pmatrix} L_{1,1} & \cdots & L_{1,j} \\ \vdots & \ddots & \vdots \\ L_{i,1} & \cdots & L_{i,j} \end{pmatrix}$$ (Equation S1)

$M_{i,j}$: i^{th} material (kg/m²) in the jth membrane design configuration. The total materials (kg/1000 m²) needed to produce the membrane configurations j are obtained by summing all the material columns in the life cycle inventory matrix (L) i.e., $\sum_{i,j}^{6} M_{ij}$.

The life cycle emissions matrix (E) for the membrane configurations (j) was obtained as a product of a 10 by 6 matrix of unit impacts (U) of each material and the life cycle inventory matrix (L) (Equation S2).

$$E = \begin{pmatrix} U_{1,1} & \cdots & U_{1,i} \\ \vdots & \ddots & \vdots \\ U_{k,1} & \cdots & U_{k,i} \end{pmatrix} \times \begin{pmatrix} L_{1,1} & \cdots & L_{1,j} \\ \vdots & \ddots & \vdots \\ L_{i,1} & \cdots & L_{i,j} \end{pmatrix}$$ (Equation S2)

$U_{k,j}$: k^{th} impact category of the ith material

These results from matrix E correspond to the impact assessment phase of LCA, where the environmental impacts of the inventory are quantified.

SI6: MATLAB Code and Data

Life cycle analysis computational code and data files in MATLAB can be accessed on GitHub repository (https://github.com/IsaacOluk/BiocharMembraneLCA)
